# Prevention of myopia in a near-primate by supplemental indigo light suggests a hypothesis for the myopia boom

**DOI:** 10.1016/j.xcrm.2026.102999

**Published:** 2026-08-18

**Authors:** Rafael Grytz, Mustapha El Hamdaoui, Takahiro Yamashita, Shane P. D'Souza, Mahmoud Tawfik KhalafAllah, Mehlika Inanici, Richard A. Lang

**Affiliations:** 1 Department of Ophthalmology and Visual Sciences, Birmingham, AL, USA; 2 Vision Science Graduate Program, University of Alabama at Birmingham, Birmingham, AL, USA; 3 Department of Biophysics, Graduate School of Science, Kyoto University, Kyoto 606-8502, Japan; 4 Department of Architecture, University of Washington, Seattle, WA, USA; 5 Division of Pediatric Ophthalmology, Cincinnati, OH, USA; 6 Science of Light Center, Cincinnati, OH, USA; 7 Abrahamson Pediatric Eye Institute, Cincinnati Children’s Hospital Medical Center, Cincinnati, OH, USA; 8 Department of Ophthalmology, University of Cincinnati, Cincinnati, OH, USA

**Keywords:** myopia, nearsightedness, emmetropization, diopter, refraction, refractive development, ocular lens, ocular lens wavelength transmission, luminance, OPN5, OPN3, OPN4, cone opsin, OPN1SW, violet light, indigo light, μ-opic, tree shrew, *Tupaia belangeri*, LARK v.3.0

## Abstract

Today, about 2.8 billion people worldwide suffer from myopia (nearsightedness), and projections place the 2050 burden at 4.8 billion people (the “myopia boom”). Myopia results from abnormal elongation of the eye, which causes images to focus in front of the retina. Building on prior work in mouse models assessing the non-visual opsin OPN5, we test the hypothesis that insufficient exposure to short-wavelength light increases myopia susceptibility. We use the tree shrew, a near-primate model for human myopia. Our findings show that indigo light in the range of 419–446 nm, when used to supplement a warm white light-emitting diode (LED), completely suppresses myopia induced by minus-lens wear. A large body of genetic and epidemiological evidence suggests that the “myopia boom” has an environmental cause. Our findings align with this evidence and suggest that one contributing factor is the relative absence of indigo light in built environments where we spend 86% of our time.

## Highlights

• Indigo light suppresses lens-induced myopia in tree shrews
• The most protective wavelength range is 419–446 nm
• Tree shrew lens filtering shifts effective OPN5 stimulation to indigo
• Built environments may lack protective indigo wavelengths

## Graphical abstract

**Figure:**
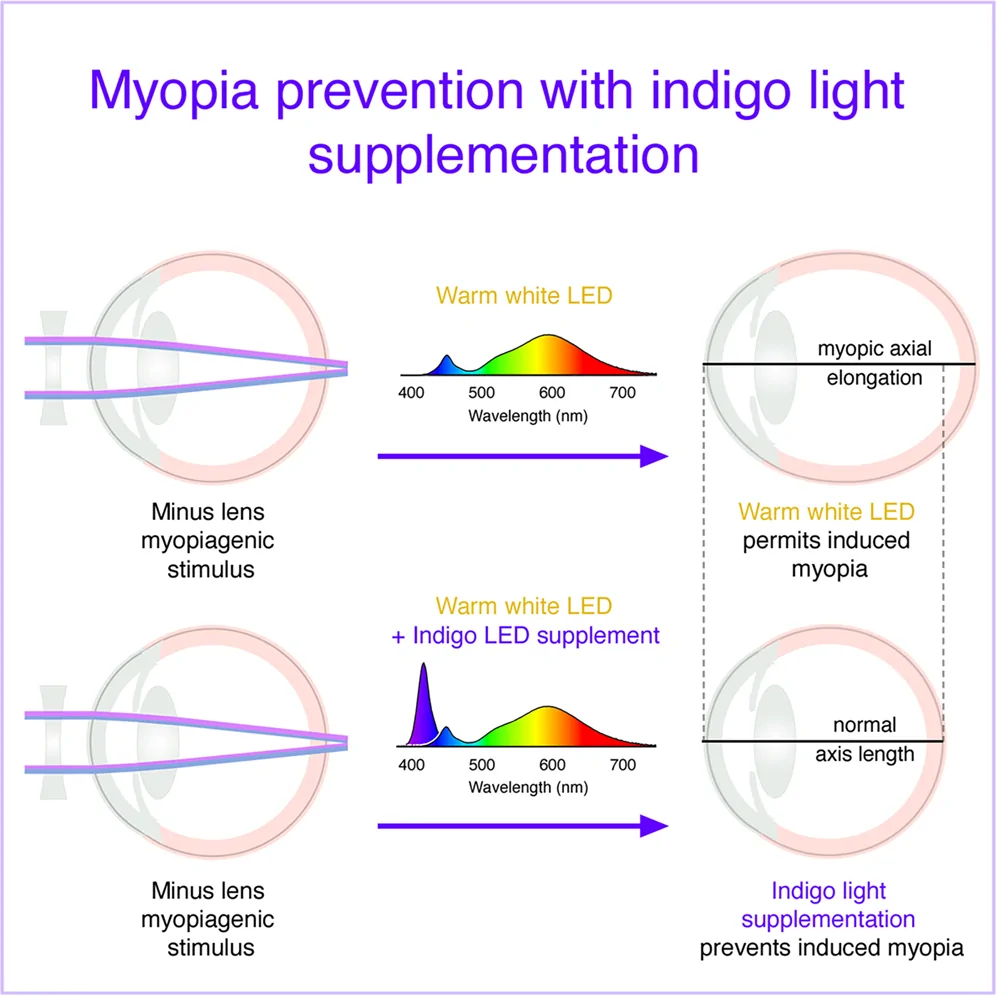

## Teaser

Grytz et al. show that indigo light supplementation prevents lens-induced myopia in tree shrews. The findings suggest that a relative absence of indigo wavelengths in built environments may contribute to the myopia boom and motivate testing indigo-enriched lighting for prevention.

## Introduction

The process of establishing perfect focus in the eye is driven by light cues. Called emmetropization,^1^ this developmental progression adjusts the axial length of the eye to ensure that images are focused on the retina. The light sensors involved are the opsin class of G protein-coupled receptor.^2^ Humans have seven opsins implicated in ocular photoreception including the cone (OPN1SW, OPN1MW, and OPN1LW) and rod (OPN2) opsins, as well as the non-canonical opsins OPN3 (encephalopsin), OPN4 (melanopsin), and OPN5 (neuropsin). These have photon absorption spectra distributed over the 350–700 nm wavelength range. Loss-of-function mutations in mouse models have indicated a role for all opsins in establishing perfect focus: this includes the rod^3^ and cone^4^^,^^5^ opsins as well as OPN3,^6^ OPN4,^7^^,^^8^ and OPN5.^9^ OPN5 is of special interest because it has the shortest wavelength response and is required for light-dependent suppression of experimentally induced myopia in mouse models.^9^ Analysis performed in the mouse models has also shown that *Opn5* is expressed in retinal ganglion cells (RGCs).^10^^,^^11^ Optimal stimulation of OPN5 occurs within the 360–370 nm^12^^,^^13^ range. These wavelengths are abundant in sunlight but largely absent from modern built environments because they are not emitted by standard lighting and are filtered out by modern glazing.^14^^,^^15^^,^^16^ Furthermore, time spent indoors is a risk factor for myopia, while time spent outdoors is protective.^17^ This confluence of information has led to the hypothesis^9^^,^^18^ that the “myopia boom”^19^^,^^20^ may be driven partly by insufficient stimulation of OPN5 (and perhaps other short-wavelength opsins) because we spend 86% of our time^21^ in built environments that limit our exposure to short-wavelength photons.

## Results

### UVA light did not effectively suppress myopia in a near-primate model

**Figure 1:**
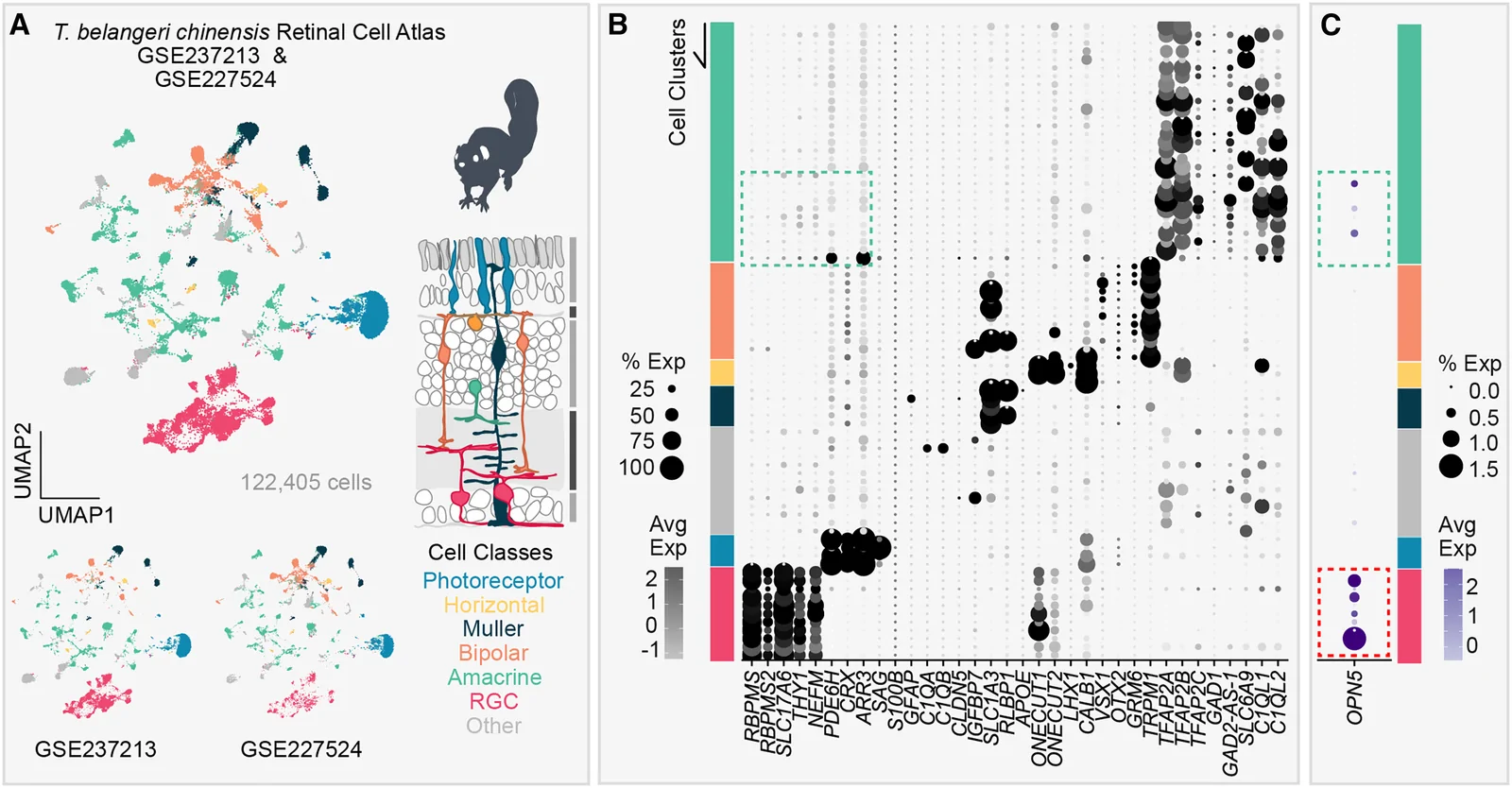
Expression of *OPN5* in ganglion cells of the tree shrew retina (A) scRNA-seq UMAPs of retinal cells from the *Tupaia belangeri chinensis* datasets GSE237213^24^ and GSE227524,^23^ either integrated (top) or individually (lower UMAP plots). Color-coding and a retinal lamination schematic depict the photoreceptor (blue), horizontal (yellow), Müller glia (black), bipolar (orange), amacrine (green), ganglionic (red), and “other” (gray) cell classes. The retinal ganglion cell (RGC) cluster (red) is at the lower edge of the UMAP plots. (B) Dot plot depicting the proportion (as a percentage according to the dot size scale) of the cell class (vertical column color-coding as in A) that expresses a class-specific marker gene (named on the horizontal axis), and the average expression level of that gene within the class (greyscale dot shading). (C) Dot plot depicting the low but detectable expression of *OPN5* (violet dot size and shading scale) across cell class clusters. Most *OPN5* expression is found in RGCs (red color-coding and dashed red box). The low levels of OPN5 expression found in the amacrine cell clusters (green cluster and dashed green box) may be attributable to nuclear doublets, and the proportion of markers for other cell types detected is shown in the leftmost green dashed box.

Prompted by these findings, we tested the activity of short-wavelength light in the suppression of induced myopia in the tree shrew *Tupaia belangeri*, a small diurnal mammal that is closely related to primates and a model for human myopia.^22^ Re-analysis of single-cell sequencing data from tree shrew retina^23^^,^^24^ indicates that, as might be expected,^10^^,^^11^ *OPN5* is expressed within cell clusters identified as RGCs according to cell type marker genes (Figure 1). Expression of *OPN5* in tree shrew RGCs suggested that UVA light might have an activity in myopia suppression similar to that observed in mice.^9^

To induce myopia in the tree shrew, animals were fitted with spectacles at 24 days after the eyes opened (24 days of visual experience [DVE]) (Figure S1), a juvenile age when they are most susceptible.^25^ In experimental animals, the spectacles incorporated a −10 Diopter (D) lens on one side to induce myopia in one eye, while the other eye served as an internal control with no lens. Normal control animals were not fitted with spectacles. Refraction and axial length were measured every 3 days in fully awake animals (Figure S1A). The −10 D lens induced axial elongation, a response that is reliable and reaches a high level of myopia in 35 days.^22^ This induced myopia model was intentionally selected because it is a strong myopiagenic stimulus that activates rapid axial elongation at the most susceptible age.

**Figure 2:**
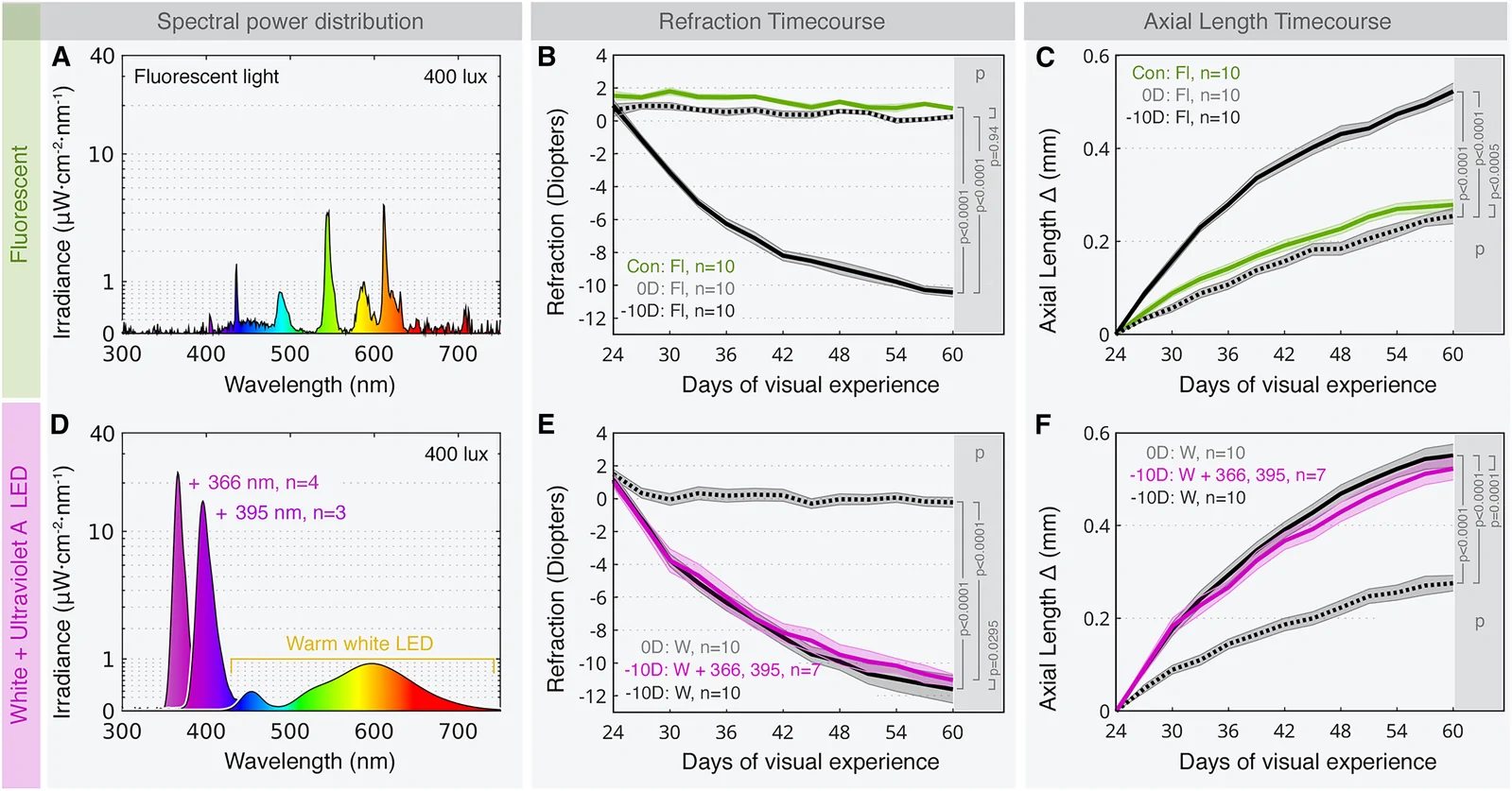
UVA supplementation does not suppress induced myopia in tree shrews (A) Chart showing spectral power output from the tree shrew colony fluorescent lights. (B and C) Charts showing refraction (B) and axial length (C) for eyes in fluorescent lighting of normal control animals (Con: Fl, *n* = 20, green trace) and for animals with spectacles and eyes with no lens (0 D: Fl, *n* = 10, dashed black trace) or with a −10 D lens (−10 D: Fl, *n* = 10, solid black trace). (D) Chart showing the spectral power output from a warm white LED (orange bracket) and the supplemental UVA light with peaks at 366 and 395 nm. Sizes for the animal cohorts for each wavelength are indicated. (E and F) Charts showing refraction (E) and axial length (F) for eyes in warm white LED lighting with no lens (0 D: W, *n* = 10, dashed black trace), with a −10 D lens (W, *n* = 10, solid black trace), or with UVA supplementation and a −10 D lens (−10 D: W + 366, 395, *n* = 7, pink trace). For (B), (C), (E), and (F), animals of both sexes were used. In (B), (C), (E), and (F), data are presented as mean ± SEM.

Like humans, tree shrews are typically born hyperopic, with a refractive error larger than 0 D. During normal development, the axial length of the eye slowly increases, actively guided by light stimuli to achieve and then maintain emmetropia (zero refractive error). In juvenile animals exposed to standard fluorescent colony lighting (Figure 2A), we observed this normal refractive development from DVE24 to DVE60 in untreated animals (Figure 2B, green trace) and for eyes fitted with spectacles with no lens (Figure 2B, black dashed trace). As expected, eyes treated with a −10 D lens developed high levels of myopia by DVE60 (Figure 2B, black trace). From DVE24 to DVE60, axial length increased in both normal control animals (Figure 2C, green trace) and in those with spectacles but no lens (Figure 2C, black dashed trace). Eyes with a −10 D lens showed accelerated axial elongation over and above the normal elongation rate (Figure 2C, black trace). This is an induced myopia.

To modify the wavelengths of light that tree shrews received during myopia induction, we designed and built ambient lighting units that included a standard “warm white” light-emitting diode (LED) as a primary photon source (Figure S2). These are typical of the commoditized 3,000 Kelvin color temperature LEDs used throughout the lighting industry. These combine the Nobel Prize- winning^26^ energy-efficient blue LED (typically emitting at 450–460 nm) with layers of phosphors that are excited by blue photons and emit in the green, yellow, and red ranges. Switching from fluorescent (Figures 2A–2C) to warm white LED lighting (Figure 2D, warm white LED) did not alter the relative refractive and axial length development of the animals fitted with spectacles, where eyes with a −10 D lens still developed high levels of myopia (Figure 2E, black trace) and accelerated axial elongation (Figure 2F, black trace).

In a first series of experiments, we determined whether LEDs centered at 366 and 395 nm providing supplemental UVA (defined as wavelengths from 315 to 400 nm) (Figure 2D) influenced induced myopia. These wavelengths were chosen because they stimulate OPN5 and, based on work performed in the mouse model,^9^ might be expected to suppress induced myopia. In the mouse analysis, UVA light was found to be most effective for myopia suppression if it was delivered for 3 h just before or just after colony lights were turned off. This lighting protocol was designed to mimic a natural dusk and recreate the high violet-to-blue wavelength ratio that occurs during nautical twilight (sun angles between −6° and −12°). However, because mice are nocturnal animals and dusk is the beginning of their active phase, in the tree shrew analysis described here, we shifted the UVA light exposure to dawn, the beginning of the tree shrew active phase, and exposed for 2 h before and 2 h after the white light was turned on (Figure S1). These lighting parameters underscore the role of OPN5 in photoentrainment of the retinal circadian clock^10^ and of circadian clock involvement in refractive development.^27^

In white LED lighting, control eyes with no lenses showed normal refraction and axial length development (Figures 2E and 2F, black dashed trace). Eyes with a −10 D lens showed the expected development of high myopia (Figure 2E, black trace) and exaggerated axial elongation (Figure 2F, black trace). UVA light supplementation (Figure 2E, pink trace) did not substantially change the induced myopic refraction (Figure 2E, pink trace) and axial length (Figure 2F, pink trace). These findings contrast with the analysis performed in the mouse model where UVA wavelengths effectively suppressed an induced myopia.^9^

### The tree shrew ocular lens is a UVA prefilter

**Figure 3:**
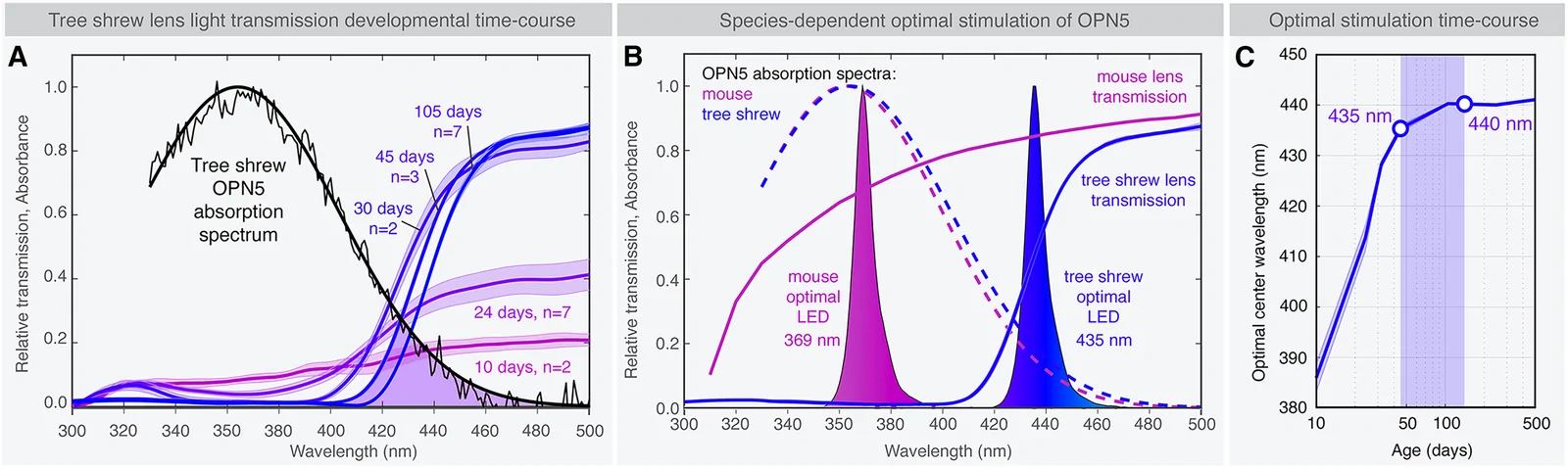
Tree shrew OPN5 absorption and ocular lens transmission characteristics (A) Absorption spectrum for OPN5 (black trace and fitted curve) overlaid on the wavelength transmission curves (colored traces) of the tree shrew ocular lens at different ages after birth (10, 24, 30, 45, and 105 days). The region of overlap between the OPN5 absorption curve and the lens transmission at 45 days of age is shaded in indigo. For wavelength transmission curves, data are presented as the mean ± SEM. (B) Chart showing the OPN5 absorption curves for mouse (dashed violet line) and tree shrew (dashed blue line), the mouse ocular lens light transmission curve (violet trace), and the lens light transmission curve for juvenile tree shrews averaged at 45 days. The colored peaks are the LED output spectra calculated to be optimal for the stimulation of OPN5 in the mouse (purple) and tree shrew (indigo). In this analysis, animals of both sexes were used. (C) Chart showing how the optimal wavelength for stimulation of OPN5 in the tree shrew changed subtly over developmental time.

We considered the possibility that the failure of UVA light to effectively suppress myopia in the tree shrew could be explained by a shift in the absorption spectrum of tree shrew OPN5. However, experimental assessment (Figures S3A–S3D) showed that the absorption spectrum peaked at 360–370 nm (Figure 3A), very close to that observed for mice.^12^^,^^13^ We then considered whether the distinct response to UVA in the mouse and tree shrew might be explained by the light transmission characteristics of the ocular lens. It has been established that the mouse ocular lens transmits UVA light.^28^ By contrast, available data suggest that the human lens does not efficiently transmit wavelengths below 400 nm and that there may be progressive absorption of shorter wavelengths with developmental age.^29^^,^^30^^,^^31^^,^^32^^,^^33^^,^^34^ To assess the transmission characteristics of the tree shrew lens, we used a wide-spectrum light source and a calibrated spectrometer (Figure S2D). Measurements of transmission were conducted immediately after enucleation, using tree shrew lenses, from 10 to 105 days of age. This showed (Figure 3A) that as with the human lens, there is a developmental progression in the transmission characteristics. The transmission curve changes shape in a complex manner over developmental time, but, in general, wavelengths below 400 nm are transmitted more in younger animals (10 and 24 days of age) (Figure 3A). While the lens transmission below 400 nm diminishes in the day 10–30 interval, it increases markedly above 400 nm and remains high thereafter (Figure 3A). This means that when the eyelids of tree shrews open at about day 21, the ocular lens transmission profile shifts toward longer wavelengths.

While the OPN5 absorption spectrum (Figure 3A) was practically identical in mice and tree shrews (Figure 3B, violet and indigo dashed traces), the lens transmission differed substantially (Figure 3B, violet and indigo traces). When the absorption curve for OPN5 is overlaid on the ocular lens transmission data (Figure 3A, black trace), it is apparent that stimulation of retinal OPN5 in maturing tree shrews can still be elicited by photons above 400 nm (Figure 3A, indigo shaded region). Calculations that take lens prefiltering into account (see STAR Methods) show that the optimal LED peak wavelength for retinal OPN5 stimulation in the mouse versus juvenile tree shrew are distinct (369 nm for mouse and 435 nm for juvenile tree shrew; Figure 3B) and that the optimal wavelength for OPN5 stimulation shifts subtly over developmental time (from 435 to 440 nm; Figure 3C). This finding suggested that indigo wavelengths might have the same myopia suppression activity in the tree shrew as UVA wavelengths in the mouse.

### Indigo light completely prevents induced myopia in the tree shrew

**Figure 4:**
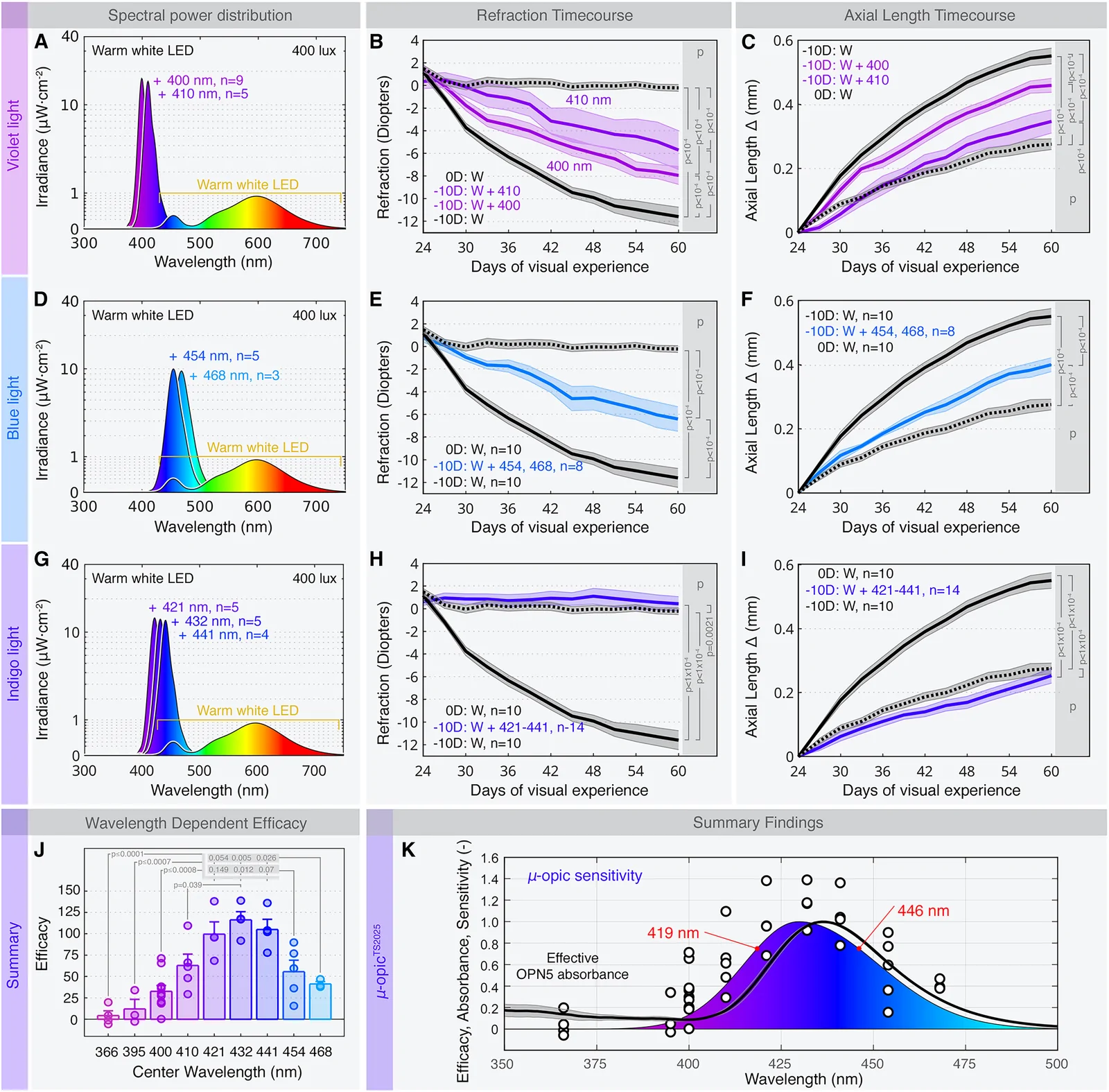
Suppression of induced myopia by short-wavelength light is wavelength sensitive (A) Spectral power output from a warm white LED and supplemental violet light, with peaks at 400 and 410 nm. Sizes of the animal cohorts for each wavelength are indicated. (B and C) Refraction (B) and axial length (C) for eyes in warm white LED lighting with no lens (0 D: W, dashed black trace), with a −10 D lens (−10 D: W, solid black trace), with 400-nm supplementation and a −10 D lens (−10 D: W + 400, dark violet trace), or with 410-nm supplementation and a −10 D lens (−10 D: W + 410, light violet trace). (D) Spectral power output from a warm white LED and supplemental blue light with peaks at 454 and 468 nm. The sizes of animal cohorts are indicated. (E and F) Same as in (B) and (C) but for 454- and 468-nm supplementation. (G) Chart showing the spectral power output from a warm white LED and the supplemental indigo light, with peaks at 421, 432, and 441 nm. The sizes of animal cohorts are indicated. (H and I) Same as in (B) and (C) but for 421-, 432-, and 441-nm supplementation. (J) Histogram showing the efficacy of supplemental wavelengths in suppressing induced myopia according to measurements of refraction and axial length at DVE60. Colored dots show data for individual animals, and the bars show the average with standard error. (K) Chart showing the μ-opic metric (violet to blue shaded curve) describing the spectral sensitivity of short-wavelength light in suppressing experimental myopia in the tree shrew when supplementing a warm white LED. The black circles show the efficacy data from individual animals. The overlaid black trace, with shading representing the standard error, shows the effective absorption of OPN5 (adjusted for lens prefiltering) and the strong correspondence with the μ-opic. The wavelengths 419 and 446 nm that represent the 75% peak height for the μ-opic are highlighted in red. For all data shown, animals of both sexes were used. In (B), (C), (E), (F), (H), (I), and (J), charts show the average measurement at each time point and the standard error as a shaded region. *p* values are shown on the gray shaded region to the right of the chart, and data are presented as mean ± SEM.

To test this, we performed additional experiments using warm white LED lighting supplemented with LEDs emitting narrow-band light with peak wavelengths over the 400–468 nm range (Figures 4A–4D and 4G). The charts show violet LEDs (400 and 410 nm, Figures 4A–4C) separately but aggregate the data for blue (454 and 468 nm, Figures 4D–4F) and indigo LEDs (421, 432, and 441 nm, Figures 4G–4I). Violet wavelengths had a modest, wavelength-dependent effect on suppression of myopia: for 400 nm, myopic refraction shifted from −11.6 D to −8.0 D at DVE60 (Figure 4B) and, for 410 nm, to −5.7 D (Figure 4B). Similarly, violet wavelengths suppressed induced axial elongation, such as in the case of 410 nm, to a length that was close to control eyes (Figure 4C). Blue wavelength supplementation (Figure 4D) also produced an intermediate suppression effect, with a DVE60 refraction of −6.4 D rather than −11.6D (Figure 4E) and an axial length closer to that of control eyes (Figure 4F). By contrast, indigo wavelength supplementation (Figure 4G) completely suppressed refractive change in response to the −10 D lens and produced eyes with a DVE60 refraction of 0.4 D, almost identical to that of controls (Figure 4H, indigo versus dashed trace). Indigo wavelength supplementation also eliminated the exaggerated axial elongation of induced myopia and resulted in a slightly reduced axial length change compared with controls throughout the DVE24–60 time course (Figure 4I). Indigo light supplementation in these analyses was at 250 μW⋅cm^−2^⋅nm^−1^ (4.4 × 10^14^ photons⋅cm^−2^⋅sec^−1^), a level designed to elicit a robust biological response. However, we also showed that a lower supplementation level of 50 μW⋅cm^−2^⋅nm^−1^ (1.0 × 10^14^ photons⋅cm^−2^⋅sec^−1^) produced a complete suppression of induced myopia (Figure S4).

In control eyes with no lens, there was a limited consequence of high-level, short-wavelength light supplementation on either refraction or axial elongation. UVA, violet, and blue supplementation resulted in DVE60 refraction of less than 1 D (Figure S5A). High-level indigo light, the most effective wavelength for myopia suppression, resulted in a DVE60 refraction of 2.2 D (Figure S5A). Similarly, eyes with no lens that received short-wavelength supplementation showed only minor changes in axial length (Figure S5B). These effects on the control eye were diminished when using low-level indigo light supplementation (Figures S5C and S5D).

These data show that in a near-primate model of induced myopia, short wavelength light supplementation can completely subvert an aggressive myopiagenic stimulus. Expressing refraction and axial length at DVE60 as a measure of efficacy (100% efficacy being complete prevention of abnormal refractive shift or axial length change) against peak wavelengths showed that suppression of myopia with supplemental short-wavelength LED light is exquisitely wavelength sensitive (Figure 4J). Notably, the indigo light that provides 100% efficacy in myopia suppression is poorly represented in the spectral power distribution of the fluorescent lights (Figure 2A) and warm white LEDs (Figure 2D) that are mainstays of the lighting industry.

As a means of quantifying the myopia prevention potential of illumination devices and illuminated environments, we developed the μ-opic metric (Figure 4K). This metric represents the spectral sensitivity of supplemental short-wavelength light in suppression of myopia. The μ-opic was derived empirically from the tree shrew efficacy data obtained in this study and is closely related to the α-opic metrics^35^ that are used to understand how light affects non-visual responses and as an aid in lighting design. Though the μ-opic is defined by myopia suppression activity, it closely follows the shape of the effective OPN5 absorption, as modified by the prefiltering activity of the ocular lens (Figure 4K, compare shaded μ-opic curve with black trace). The μ-opic sensitivity spectrum has a slightly asymmetric shape and extends further into the blue compared with the violet and UVA spectra (Figure 4K). The upper quartile values of the μ-opic (red numbers in Figure 4K) showed that short wavelengths in the indigo range of 419–446 nm are the most effective supplement for suppressing myopia.

### Simulation of an artificially lighted environment designed for myopia prevention

**Figure 5:**
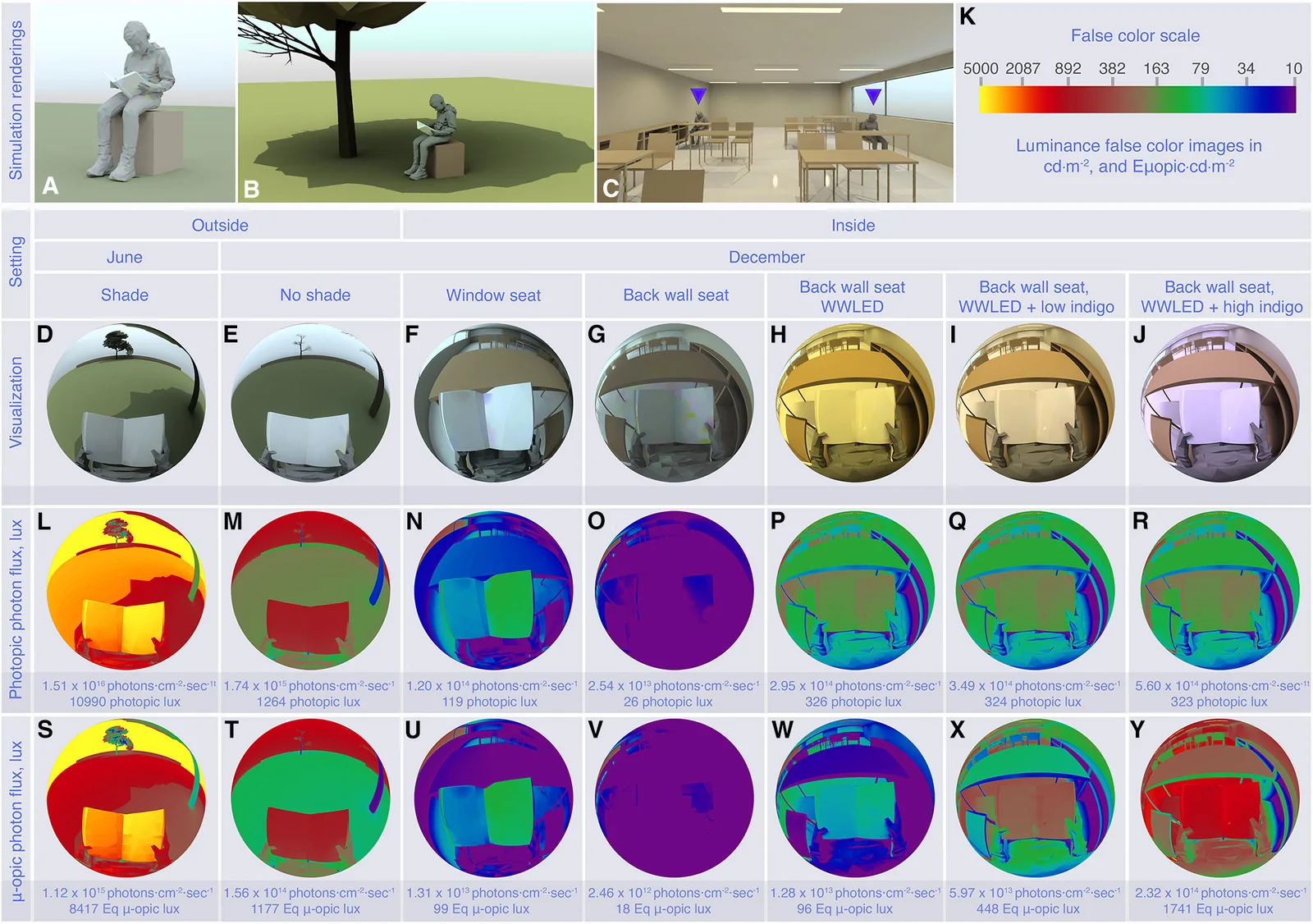
Simulation of a lighted room with wavelengths for myopia prevention (A–C) Renderings representing the seated child subject (A), the outside shaded location (B), and the inside locations (C) adjacent to the window (right arrowhead) and at the back wall (left arrowhead) that were quantified in the simulation. (D–J) Visualization of the subject view in the different simulation locations and lighting conditions as in the labeled columns: June outside in the shade (D), December (E–J), outside, no shade (E), inside (F–J), at the window seat (F), at the back wall seat (G–J), with warm white LED lighting (WWLED) (H), with WWLED + low level indigo LED (I), or with WWLED + high level indigo LED (J). (K) False color scale showing candela per meter squared (cd⋅m^−2^). (L–Y) Illustration of the photopic (L–R) and μ-opic (S–Y) luminance for the same settings, based on the false color scale. The total photon flux, photopic lux, μ-opic photon flux, and equivalent μ-opic lux (L–Y) were calculated for the views shown in (D)–(J).

Derivation of the μ-opic creates the opportunity to assess simulated illuminated environments for myopia prevention. To do this, we used LARK v.3.0 multispectral lighting simulation^16^ for a model classroom (Figure 5), a setting chosen because children are a good myopia prevention target.^36^^,^^37^ LARK computes illumination via a 9-channel version of the RADIANCE lighting simulation engine.^38^ Comparisons with physical measurements showed that LARK was able to predict values within acceptable error margins.^39^^,^^40^ Here, we used LARK for the simulation of photopic (visual wavelengths) and μ-opic illumination at the eyes of a seated child (Figure 5A). Figures S7 and S8 illustrate the spectral properties of light sources and building surfaces. We performed this analysis for Seattle in June and December outside (Figure 5B) and inside a windowed classroom with chairs and desks (Figure 5C). Within the classroom, we simulated locations either adjacent to or distant from double-paned energy-efficient south-facing windows with low-emissivity glass (Figure 5C, arrowheads). Electric lighting simulation included a warm white LED (Figure 5, WWLED) alone (Figures 5H, 5P, and 5W) or enhanced with either low (Figures 5I, 5Q, and 5X) or high (Figures 5J, 5R, and 5Y) levels of indigo light that reflect the levels used in animal experiments. The electric light fixtures were downward-facing linear LED lamps fitted with typical optical components that control glare and light distribution. A common classroom layout of indoor luminaires was chosen that provided 326 photopic lux at eye level for the model child.

In animal experiments, 400 lux WWLED light was supplemented with indigo light at a high level (250 μW⋅cm^−2^⋅nm^−1^, 4.4 × 10^14^ μ-opic photons⋅cm^−2^⋅sec^−1^) or low level (50 μW⋅cm^−2^⋅nm^−1^, 1.0 × 10^14^ μ-opic photons⋅cm^−2^⋅sec^−1^). The relative spectral irradiances from the animal experiments were used to model the luminaires of the classroom. However, the overall light intensity was adjusted so that a similar illuminance at eye level (323–326 photopic lux) was maintained across simulated scenarios that used electrical lighting. This approach shows that changes in spectral composition and the proposed myopia prevention activity can be targeted without modifying the absolute illuminance for visual function. High-level indigo was used for most of the tree shrew analysis (Figure 4), but the lower level is a more practical supplementation as it provides better color rendition. Notably, the lower level of indigo supplementation still provides 100% efficacy in suppression of induced myopia (Figure S5).

In the simulation, the subject’s view of a book and the classroom is the result of light emitted from fixtures and reflected from objects (luminance). A visualization of the scene is presented in color tones (Figures 5D–5J), while a false color scale (Figure 5K) shows luminance weighted for the wavelength ranges used for vision (Figures 5L–5R, candelas/m^2^) and for myopia prevention (Figures 5S–5Y, equivalent μ-opic candelas/m^2^). Two other measures are listed below each color map—the illuminance (a measure of light on a surface or in a plane, in photopic lux or equivalent μ-opic lux) and the photon flux (total or μ-opic photons⋅cm^−2^⋅sec^−1^) (Figures 5L–5Y). Because it is the lowest lighting level known to be effective for myopia suppression (Figure S5), we used the μ-opic photon flux of the low-level indigo supplementation (1.0 × 10^14^ μ-opic photons⋅cm^−2^⋅sec^−1^) as a benchmark for comparison.

Simulation of daylight on a summer day (Figure 5S) in the shade or on a winter day (Figure 5T) showed sufficient μ-opic photon flux compared with the benchmark (11.2-fold and 1.56-fold higher, respectively). The analysis emphasizes the disadvantage for the proposed myopia prevention of living inside: the window and back wall locations showed a 7.6-fold and 41-fold reduction in μ-opic photon flux, respectively, compared with the preventive benchmark (5U and 5V). The simulation also showed that at the back wall position, warm white LEDs can provide a good level of photopic illumination useful for visual function (Figure 5P; 2.95 × 10^14^ photons⋅cm^−2^⋅sec^−1^/326 photopic lux). However, illumination from warm white LEDs within the μ-opic (1.28 × 10^13^ photons⋅cm^−2^⋅sec^−1^/96 Eq μ-opic lux) was low. By contrast, illumination from warm white LEDs supplemented with low-level indigo could satisfy both the photopic (Figure 5Q) and μ-opic (5.97 × 10^13^ photons⋅cm^−2^⋅sec^−1^/448 Eq μ-opic lux) requirements. These findings illustrate how indigo-enhanced lighting can overcome, for the proposed myopia prevention, the limitations imposed by inside living and illuminating with warm white LEDs.

## Discussion

A large body of genetic and epidemiological evidence has indicated that the “myopia boom”^19^^,^^20^ is likely caused by an environmental factor, rather than through the propagation of risk alleles.^41^^,^^42^^,^^43^ It has also been established that time spent outside is beneficial in preventing myopia,^44^^,^^45^ while time spent inside increases the risk of the disease.^46^ The current analysis is consistent with these data and suggests that the abundance of 419–446 nm photons in sunlight may explain why outside time reduces the myopia risk. Similarly, the relative absence of these wavelengths in built environments may explain why inside time is a risk factor.

This analysis determined empirically the most effective short wavelengths for myopia suppression in the tree shrew model. The tree shrew was chosen for this investigation because it is a diurnal near-primate and, thus, a valuable model for defining myopia prevention conditions for humans. This work was motivated by an analysis of the non-visual opsin OPN5 in the mouse showing that UVA light in the 360–400 nm wavelength range suppressed an induced myopia.^9^ This was a provocative finding because it suggested a specific molecular pathway through which short-wavelength light could influence refractive development and myopia susceptibility.^47^^,^^48^^,^^49^^,^^50^^,^^51^ Further, this finding is consistent with epidemiological evidence suggesting that the “myopia boom”^19^^,^^20^ is caused by an environmental factor and that sunlight, with its abundant short-wavelength photons, is protective.^52^ This analysis has defined several conclusions and future questions as follows.

OPN5 is highly conserved within Mammalia,^53^ and, according to molecular analysis, mouse and human OPN5 are activated most efficiently by 360–370 nm photons within the UVA band.^12^^,^^13^ Despite prior analysis in mice,^9^ UVA failed to suppress induced myopia in the tree shrew. Differences in absorption characteristics of tree shrew and mouse OPN5 (they were essentially identical) could not explain why UVA failed to suppress myopia in the tree shrew. However, further investigation showed that the tree shrew ocular lens, in contrast to the mouse ocular lens,^28^ does not efficiently transmit wavelengths below 400 nm. The tree shrew lens could, thus, act as a prefilter, restricting the wavelengths that can reach OPN5 within RGCs.^9^^,^^54^ Consistent with this, the indigo wavelengths calculated to be optimal for stimulating OPN5 through the ocular lens were shown to be highly effective at suppressing induced myopia in a near-primate. It has been established that the human lens also shows poor transmittance of UVA wavelengths and that there is a developmental stage dependence, with younger lenses showing greater transmittance of violet and indigo light.^29^^,^^30^^,^^31^^,^^32^^,^^33^^,^^34^ The conservation of this feature of the human and tree shrew lens suggests that indigo supplementation should be a high-priority wavelength range to test for myopia prevention in humans.

Here, we defined the μ-opic, a metric that quantifies the efficiency of short-wavelength supplemental light in myopia prevention. The μ-opic spectral sensitivity is broad and slightly asymmetric, suggesting that it may reflect the activity of multiple opsins besides OPN5. These could include OPN3, and though the absorption spectrum is not well-defined, vertebrates show the peak absorption at 470 nm,^55^ consistent with blue light stimulation of OPN3-dependent responses.^56^^,^^57^ Analysis in the mouse models has indicated that OPN3 can influence refractive development as well as the expression of genes that respond to refractive mismatch.^6^^,^^58^ OPN4, with its absorption peak at 490 nm, also tends to be activated by wavelengths within the μ-opic. The refractive development and induced myopia phenotypes of mice with OPN4 loss-of-function^7^^,^^8^ are consistent with a role for this opsin. The absorption spectrum of the blue cone opsin OPN1SW also falls within the μ-opic (we confirmed that tree shrew OPN1SW absorbs at 440 nm; Figure S3E).^59^ Though there is evidence implicating the red light-responsive opsin OPN1LW in myopia etiology in humans,^60^ there is limited evidence for involvement of OPN1SW. In a mouse model, cone transducin-α (encoded by *Gnat2*) loss-of-function did not change refractive development but increased susceptibility to induced myopia^4^ and mediated the protective effect of short-wavelength light.^61^ Mutation of *Gnat1*, encoding the rod photoreceptor transducin-α, results in abnormal refractive development and a failure to respond to induced myopia.^3^ This establishes the crucial role of rod photoreceptors in emmetropization. Rod photoreceptors would be expected to respond to light wavelengths within the μ-opic. Interestingly, the choroid is also sensitive to wavelengths within the μ-opic, where violet light was found to directly regulate choroidal perfusion.^62^ The choroid is known to be involved in eye growth regulation^63^ and has been proposed to play a key role in myopia suppression by short-wavelength light.^51^

Recent work in the tree shrew shows that narrow-band blue light (in the absence of long wavelengths) can lead to a slow drift toward myopia in otherwise unmanipulated animals.^64^ Here, we show that indigo light can suppress induced myopia when used in combination with white light. Together, these studies suggest that full-spectrum light may be important for effective myopia suppression. Furthermore, indigo light supplementation had a profound effect on eyes with lens-induced high myopia but a small effect on control eyes with no lens. This suggests that the effect of indigo light is conditional and most effective when a myopiagenic stimulus is present. While high- and low-level indigo light completely suppressed induced myopia, only high-level indigo light could cause a small amount of hyperopia in control eyes. Further research is needed to establish light intensities and treatment durations that are effective for myopia suppression without the risk of inducing hyperopia.

Data generated in mice showed that short-wavelength violet light loses its effectiveness in myopia suppression when used all day, suggesting that diurnal variation in spectral cues may also be important for myopia suppression.^9^ When combined with prior analyses, the current study supports the hypothesis that myopia resistance results from the exposure to both short- and long-wavelength light as presented with natural variations of the sunlight spectrum.

Use of the μ-opic metric to assess simulated illuminated environments illustrated the disadvantage of indoor living and the deficiency of warm white LEDs in providing indigo wavelengths for myopia prevention. The μ-opic can also be used for designing illuminated environments that deploy the often-preferred warm white light but also provide the proposed myopia prevention benefit of indigo light. Furthermore, the μ-opic can be used to guide the incorporation of glazing that transmits shorter-wavelength light with higher efficiency and potentially provides an advantage for myopia prevention. We expect that the μ-opic metric will be refined after human studies of indigo light myopia prevention have been completed, but also anticipate, given the high evolutionary conservation between tree shrews and humans, broad opsin absorption spectra, and knowledge of natural sunlight spectra, that more precision in the μ-opic is not essential for effective deployment.

Several testable predictions with translational implications emerge from this study. (1) Increasing daytime outdoor exposure is supported as a strategy to reduce myopia; our findings identify indigo light as one candidate component of the outdoor signal. (2) Indoor lighting spectra that raise μ-opic exposure in the 419–446 nm band should be evaluated as a practical myopia prevention strategy in children. (3) Because short-wavelength-attenuating (“blue-blocking”) spectacles reduce indigo light transmission to the eyes, widespread daytime use in children could plausibly increase the risk of myopia. If these hypotheses are confirmed in humans, spectral modifications to built environments could contribute to reducing the growing burden of myopia.^19^^,^^20^

### Limitations of the study

This study demonstrates that indigo light in the range of 419–446 nm could suppress experimentally induced myopia in the tree shrew, a near-primate. Though this is a valuable experimental model, it does not fully mimic the natural history of myopia development and progression in humans. Studies that directly compare the influence of standard lighting and indigo-supplemented lighting on responses in the human eye are an obvious next step. Furthermore, though this study was initiated in response to questions about the function of OPN5 in myopia pathways, it does not directly address the mechanisms that underly this response. A comparison of the molecular changes that occur in the tree shrew eye tissues in control eyes and eyes with induced myopia, in standard and indigo-supplemented light, is likely to provide insights into these mechanisms.

## Resource availability

### Lead contact

Requests for further information, resources, and reagents should be directed to and will be fulfilled by the lead contact, Rafael Grytz (rafaelgrytz@uabmc.edu).

### Materials availability

Requests for resources and reagents should be directed to the lead contact.

### Data and code availability

• The data file TreeShrew_all_cells_integrated.RDS that is relevant to this analysis has been deposited in the Mendeley repository.
• This paper does not report original code.
• Source data are provided with this paper in the file Grytz_et_al_2026_source_data.xlsx. Any additional information required to reanalyze the data reported in this work paper is available from the lead contact upon request.

## Acknowledgments

We thank Prof. Russell Van Gelder for reading the manuscript, Prof. Robert S. Molday for the generous gift of a Rho1D4-producing hybridoma, Dr. Kazumi Sakai for technical support on the measurement of opsin absorption spectra, and Chester Calvert and Eric Worthington for manufacturing the light units, measurement tools, and tree shrew goggles. The Grytz lab acknowledges support from the National Eye Institute of the National Institutes of Health, USA (grants EY026588, EY036560, EY032633, EY003039, EY038143, and EY028666), from the EyeSight Foundation of Alabama, from Research to Prevent Blindness, and charitable donations from the Henry M Hollis Fund. The Lang lab acknowledges support from National Institutes of Health USA, the National Eye Institute (grants EY032029, EY032752, EY032566, and EY034456), the National Institute of General Medical Sciences (grant GM152641), the Emma and Irving Goldman Scholar Endowed Chair, and the Cincinnati Children’s Hospital Research Foundation Academic and Research Committee. The Yamashita lab acknowledges funding support from the Japan Agency for Medical Research and Development (AMED) CREST (grant 22gm1510007).

## Author contributions

R.A.L. and R.G. conceived the project; S.P.D. performed analysis of single-cell sequencing data; R.G. and R.A.L. designed the tree shrew analysis; R.G. derived the μ-opic; M.E.H. performed the statistical analysis; R.G., M.E.H., and M.T.K. executed tree shrew experimental work; T.Y. performed analysis of opsin absorption; M.I. performed lighting simulation analysis; R.A.L. drafted the manuscript; R.A.L., R.G., T.Y., and M.I. edited the manuscript.

## Declaration of interests

R.G. and R.A.L. are inventors on pending patents for lighting devices related to this work. R.G. is founder and CSO of Electric Indigo, a UAB startup in which UAB holds an ownership interest. Electric Indigo will pursue myopia prevention technologies based on the current analysis.

## Declaration of generative AI and AI-assisted technologies in the writing process

During the preparation of this work, the authors used ChatGPT Pro to reformat the “methods” section into the STAR Methods format. After using this tool, the authors reviewed and edited the content as needed and take full responsibility for the content of the published article.

## STAR★Methods

### Key resources table

**Table:** 

REAGENT or RESOURCE	SOURCE	IDENTIFIER
**Antibodies**		

1D4 hybridoma (anti-rhodopsin antibody)	Gift from R.S. Molday	RRID: AB_2315272

**Chemicals, peptides, and recombinant proteins**		

11-cis-retinal	Sigma-Aldrich or as referenced	CAS # 548-62-9

**Deposited data**		

Tupaia belangeri chinensis retinal single cell sequencing data, GSE237213	Hahn et al.^24^	GEO: GSE237213
Tupaia belangeri chinensis retinal single cell sequencing data, GSE227524	Xiong et al.^23^	GEO: GSE227524
TreeShrew_all_cells_integrated.RDS	This paper; Mendeley repository	https://data.mendeley.com/datasets/gbm38vntv7/1
Source data	This paper	Grytz et al. 2026 source data.xlsx

**Experimental models: Cell lines**		

HEK293S cells	ATCC	ATCC CRL-1573

**Experimental models: Organisms/strains**		

Tupaia belangeri (tree shrew)	UAB Breeding Colony	N/A

**Recombinant DNA**		

pCAGGS expression vector	Niwa et al.^65^	Addgene or constructed in-lab
Tupaia belangeri chinensis OPN5 cDNA, C-terminal 36 amino acid truncation, 1D4-tagged	This paper	NLM accession number XM_006149093

**Software and algorithms**		

Seurat v5	Seurat	https://satijalab.org/seurat/
MATLAB R2021b	MathWorks	https://www.mathworks.com/products/matlab.html
R v4.3.2	R Project	https://cran.r-project.org/
LARK v3.0 multispectral lighting simulation	Jung et al.^16^	N/A
RADIANCE lighting simulation engine	Ward^38^	N/A

**Other**		

Nidek ARK-700A auto-refractor	Marco Ophthalmic	N/A
Lenstar LS-900 biometer	Haag-Streit USA	N/A
FLAME-S-UV-VIS-ES spectrometer	Ocean Optics	Model: FLAME-S-UV-VIS-ES
PX-2 xenon light source	Ocean Optics	Model: PX-2
CC-3-UV-S cosine corrector	Ocean Optics	Model: CC-3-UV-S
74-UV collimating lens	Ocean Optics	Model: 74-UV
UV-2600 UV-visible spectrophotometer	Shimadzu	Model: UV-2600
Master HILUX-HR 1 kW halogen lamp	Rikagaku	Model: Master HILUX-HR
Custom controlled lighting units	This paper	N/A
Warm white and narrowband light emitting diodes	Custom made for this study	Spectral irradiance values in Source Data, sheet “irradiance”
PMMA -10D lens	N/A	12 mm diameter; 7.5 mm base curve

### Experimental model and study participant details

#### Animals

The use of animals in this work was approved by the University of Alabama at Birmingham Institutional Animal Care and Use Committee (protocol ID IACUC-20512). Three cohorts of animals were used for assessing the effects of supplemented short wavelength light on lens induced myopia. Animals of the first cohort were assigned randomly to the five groups shown in Figure 2: one normal control group and one experimental group in standard fluorescent colony lighting, three experimental groups in warm white LED lighting with (2 groups) and without (1 group) UVA light supplementation. Animals of the second cohort were randomly assigned to the seven experimental groups shown in Figure 4 with violet (2 groups), blue (2 groups), and indigo (3 groups) light supplementation. A third cohort was used to test the effect of low-level indigo light supplementation as shown in Figure S5. Across all cohorts, we did not assign littermates to the same group. Investigators were not blinded to allocation of animals during experiments and outcome assessments including refractive and axial measurements.

#### Tree shrew model of induced myopia

Northern tree shrews (*Tupaia belangeri*) of both sexes were obtained from the breeding colony at University of Alabama at Birmingham. At DVE21, animals were weaned and under anesthesia a dental acrylic pedestal was surgically installed.^66^ At DVE24, lightweight aluminum spectacles were clipped onto the pedestal.^66^ Beforehand, a −10D lens (PMMA, 12 mm diameter, 7.5 mm base curve) was randomly installed into the right or left side of the spectacles while the other side remained open. The PMMA lens had a transmittance of 87–93% for wavelengths between 350 nm and 750 nm. While the spectacles were fitted to each animal so that the lens was positioned close to the eye, there was a small gap (∼1 mm) between the spectacle frame and the surrounding eye tissues allowing light to reach the eye without passing through the PMMA lens. The −10D lens shifts the focal plane behind the retina, which accelerates axial elongation of the eye and induces high myopia.^22^ Starting at DVE24, refraction and axial length measurements were obtained in conscious animals using the Nidek ARK-700A infrared auto-refractor (Marco Ophthalmic, Jacksonville, FL) and the Lenstar LS-900 optical biometer (Haag-Streit USA, Mason, OH), respectively. The spectacles were removed during these measurements and reattached afterward. These measurements were performed in a darkened room in the morning (around 10 a.m.) and repeated every three days until the experimental endpoint (DVE60). Figure S1A illustrates the time course of the experiment. A constant value of 4 Diopters was subtracted from the spherical equivalent to correct for the so-called “small eye artifact”.^67^ Tree shrew specific refractive indices were used to analyze the Lenstar data and calculate axial length.^68^ While the eyes of maturing animals develop toward zero refractive error, a similar absolute target value does not exist for axial length. Consequently, the absolute values of spherical equivalent were used for further analysis, while changes from baseline were used for axial length. The refractive and axial length data can be found in Source Data (sheet “refraction_axial_length”). We did not observe any sex-dependent differences.

### Method details

#### *Tupaia belangeri chinensis* retinal atlas

##### Preprocessing

*Tupaia belangeri chinensis* single cell sequencing gene by cell matrices (GSE237213^24^ & GSE227524^23^) were downloaded and processed using either a Sketch-based or PCA-integration Seurat (v5) workflow. Matrices were merged into a single SeuratObject and subsequently normalized using default parameters for normalization (NormalizeData) and variable feature selection (FindVariableFeatures; features = 2000). To integrate the two datasets, we used a sketch-based approach (SketchData; ncells = 5000, method = “LeverageScore”), followed by scaling (ScaleData) and principal component analysis (RunPCA) on the sketch-assay. To integrate these layers, we performed integration (IntegrateLayers; k.anchors = 20, dims = 1:30) via reciprocal PCA using GSE237213 as a reference. We then performed neighbor analysis (FindNeighbors; reduction = “integrated.pca”, dims = 1:30), clustering (FindClusters; resolution = 2), and dimensionality reduction (RunUMAP; reduction = “integrated.pca”, dims = 1:30). Then, the integration and sketch-data were projected onto the non-sketched cells (ProjectIntegration; ProjectData) to identify clusters.

#### Cell class identification

To identify cell classes that correspond to *Tupaia belangeri chinensis* atlas clusters, we projected modules of canonical markers^24^ onto the integrated atlas. Clusters with mean projected-module scores greater than 0 were assigned to a cell class. When encountered, clusters with two annotations were manually inspected for gene expression of cell class markers and manually assigned to a final class based on expression. Canonical markers included for each cell class are as follows:

• Retinal ganglion cell markers = RBPMS, RBPMS2, SLC17A6, THY1, NEFM.
• Bipolar cell markers = VSX1, OTX2, GRM6, TRPM1.
• Amacrine cell markers = TFAP2A, TFAP2B, TFAP2C, GAD1, GAD2-AS-1, SLC6A9, C1QL1, C1QL2.
• Horizontal cell markers = ONECUT1, ONECUT2, LHX1, CALB1.
• Photoreceptor cell markers = PDE6H, CRX, ARR3, SAG.
• Muller glial cell markers = SLC1A3, RLBP1, APOE.
• Other markers = S100B, GFAP, C1QA, C1QB, CLDN5, IGFBP7.

#### Lighting conditions

All tree shrews were housed in a 14 h/10 h light/dark cycle with the light cycle starting at 6:30 a.m. Before the experiment started, all animals were housed in standard fluorescent colony lighting with an approximate illuminance of 400 lx at the bottom of the cage. During the experiment, tree shrews were housed individually in cages with dimensions 20″ x 24″ x 35” (width x length x height). Tree shrew groups reported in Figures 2B and 2C remained in rooms with standard colony fluorescent lighting throughout the experiment. All other groups were housed under controlled lighting conditions using custom light units made from an aluminum frame (Figure S2A). Light units consisted of LEDs producing warm white light (3000 K) and narrowband short wavelength light with peak wavelengths at 366 nm, 395 nm, 400 nm, 410 nm, 421 nm, 432 nm, 441 nm, 454 nm, and 468 nm. One light unit was placed on top of each cage (Figure S2B) and the light intensity was adjusted to 400 lx warm white light, which was supplemented with short wavelength LED light at 250 μW/cm^2^. All light measurements were obtained at the bottom of the cage using a calibrated spectrometer (FLAME-S-UV-VIS-ES, Ocean Optics, Orlando, FL) with a wavelength range of 200–850 nm. Attached to the spectrometer was an optical fiber and cosine corrector (CC-3-UV-S, Ocean Optics) pointing upwards toward the light unit during measurements. A bi-symmetric log transformation was used for plotting the spectral irradiance of each LED in Figures 2A, 2D, 4A, 4D and G; Figure S5A.^69^

For all short wavelength supplementation shown in Figure 2, narrowband light was used for 4 h in the morning starting at 6:30 a.m. To mimic the high content of short wavelengths of dawn, narrowband light was used for 2 h alone followed by 2 h in combination with warm white light. Warm white light was used for the remaining 10 h to complete the 14-h light cycle illustrated in Figure S1B. Cages with different lighting conditions were housed in separate bays (Figure S2C). Each bay was isolated from other light sources by means of blackout curtains. Most narrowband LEDs were custom made for this study. To allow the replication of our results, the spectral irradiance of each light source can be found in Source Data (sheet “irradiance”). For low-level indigo light supplementation (Figure S4) the LEDs employed had a peak output at 421 nm and were used for one hour at dawn, starting at 8 a.m. and finishing at 9 a.m., with warm white light turning on at 8:30 a.m.

#### Measurement of absorption spectra of tree shrew opsins

Recombinant protein of *Tupaia belangeri chinensis* OPN5 and OPN1SW were obtained in HEK293S cells. To improve the expression level of OPN5, the cDNA (NLM accession number XM_006149093) was modified to produce a C-terminal 36 amino acid truncation. This C-terminal deletion produced higher expression levels of human OPN5 in a previous study.^12^ The C-terminally truncated cDNA was tagged with the 1D4 epitope of bovine rhodopsin and was introduced into the mammalian expression vector pCAGGS.^65^ The plasmid was transfected into HEK293S cells using the calcium-phosphate method (this cell line was authenticated by short tandem repeat profiling – and tested negative for mycoplasma contamination by PCR). 5 μM 11-*cis* retinal was added to the medium 24 h after transfection and the cells were kept in the dark until they were collected 48 h after transfection. The reconstituted pigments were extracted from cell membranes with 1% dodecyl maltoside (DDM) in Buffer A (50 mM HEPES, 140 mM NaCl, pH6.5) and were purified using Rho1D4-conjugated agarose. The purified pigments were eluted with 0.02% DDM in Buffer A containing the synthetic peptide that corresponds to the C terminus of bovine rhodopsin. Absorption spectra of OPN5 were recorded with a UV-visible spectrophotometer (UV-2600, Shimadzu). The OPN5 sample was kept at 0°C using a cell holder equipped with a circulation system that uses temperature-controlled water. The OPN5 absorption spectra were measured in the dark and after UV light (360 nm) irradiation with light through a UVD-36C glass filter from a 1 kW halogen lamp (Master HILUX-HR; Rikagaku). The difference spectrum of OPN5 was obtained by subtracting the spectrum before irradiation from that measured after UV light irradiation. Further calculations performed according to the Lamb and Govardovskii method,^70^^,^^71^ as shown in previous studies,^12^^,^^72^ identified the absorption spectrum of the 11-*cis*-retinal and all-*trans*-retinal bound states of tree shrew OPN5. The tree shrew and mouse OPN5 absorption spectra^12^ can be found in Source Data (sheet “OPN5_absorption”).

#### Measurements of light transmission of tree shrew ocular lens

To assess which short wavelengths can reach the retina in tree shrews, we measured the ocular lens transmission of tree shrews at different ages using an optic fiber-coupled measurement device shown in Figure S2D. The device consists of a wide spectrum light source (pulsed xenon lamp, 220-750 nm, PX-2, Ocean Optics) connected to an optical fiber and collimating lens (74-UV, Ocean Optics). The light was transmitted through a 2 mm diameter pin hole onto which the lens was placed immediately after enucleation. The transmitted light was measured within a light-tight housing using the same spectrometer setup detailed under section “Lighting conditions”. The wavelength-dependent transmission data was smoothed using the Savitzky-Golay filter implemented in MATLAB 2021b.

To estimate which narrowband LED light source is optimal for retinal OPN5 stimulation while accounting for the prefiltering of the ocular lens, we maximize the following cost function

(Equation 1) $$cost=\int {\bar{E}}_{e,\lambda }\left(\lambda +\lambda_{\max}-\lambda_{\max}^{opt}\right)\cdot \tau \left(\lambda \right)\cdot \bar{A}\left(\lambda \right)\;d\lambda$$

where ${\bar{E}}_{e,\lambda }\left(\lambda \right)$ represents the normalized spectral irradiance profile of a narrowband LED with peak wavelength *λ*_*max*_, *τ*(*λ*) is the transmittance of the ocular lens, and $\bar{A}\left(\lambda \right)$ is the normalized absorption spectrum for OPN5. The only unknown parameter is $\lambda_{\max}^{opt}$ representing the optimal peak wavelength of the narrowband LED for retinal OPN5 stimulation. For ${\bar{E}}_{e,\lambda }\left(\lambda \right)$ we used the spectral irradiance profile of the narrowband UVA LED light source with a peak wavelength of *λ*_*max*_ = 366 nm shown in Figure 2D.

#### Lighting simulation

We used LARK v3.0 multispectral lighting simulation^16^ for a model classroom. LARK computes illumination via a 9-channel version of the RADIANCE lighting simulation engine.^38^ Comparisons with physical measurements show that Lark was able to predict values within acceptable error margins.^39^^,^^40^^,^^73^ The normalized spectra for the illumination sources used in the simulation are described in Figure S7. The spectral properties of the major surfaces used in the simulation are shown in Figure S8.

#### The μ-opic spectral sensitivity of short wavelength light to suppress myopia

The μ-opic is an empirically derived metric inspired by the α-opic metric.^74^ Like the α-opic, the μ-opic can be used to design illuminated environments, but specifically those that are designed to prevent the development of myopia. Following the nomenclature of the α-opic, we define the effective μ-opic photobiological irradiance *E*_*μ*_ [W·m^−2^] to suppress myopia as:

(Equation 2) $$E_{\mu }=\int E_{e,\lambda }\left(\lambda \right)\cdot S_{\mu }\left(\lambda \right)d\lambda$$

where *E*_*e*,*λ*_(*λ*) [W·m^−2^·nm^−1^] represents the spectral irradiance of a light source and *s*_*μ*_(*λ*) [-] is the wavelength-dependent sensitivity or action spectrum of short wavelength light to suppresses myopia. To estimate this action spectrum, we assume that *s*_*μ*_(*λ*) has a skewed Gaussian shape and that the efficacy *ϵ*_*i*_ of a light source is proportional to its effective photobiological irradiance (2):

(Equation 3) $$\epsilon_{i}=\frac{E_{\mu ,i}}{K}=\frac{1}{K}\int E_{e,i,\lambda }\left(\lambda \right)\cdot s_{\mu }\left(\lambda \right)\;d\lambda$$

where *K* [W·m^−2^] is an unknown constant. Note that additional factors may influence *ϵ*_*i*_ [-] such as the shorth wavelength light intensity, duration, time of day, and the relative intensity of long wavelength light. However, these and other potential factors were disregarded as all animals in our study were exposed to the same conditions except the spectral irradiance *E*_*e*,*i*,*λ*_(*λ*) [W·m^−2^·nm^−1^]. We have estimated the unknown parameters of the skewed Gaussian function and *K* by minimizing the residual sum of squared errors:

(Equation 4) $$RSS=\sum_{i}{\left[\epsilon_{i}-\frac{E_{\mu ,i}}{K}\right]}^{2}=\sum_{i}{\left[\epsilon_{i}-\frac{1}{K}\int E_{e,i,\lambda }\left(\lambda \right)\cdot s_{\mu }\left(\lambda \right)\;d\lambda \right]}^{2}$$

Within Equation 4, *ϵ*_*i*_ [-] represents the efficacy of each animal experiment shown in Figure 4J and *E*_*e*,*i*,*λ*_(*λ*) [W·m^−2^·nm^−1^] the corresponding spectral irradiance combining the warm white LED and the corresponding short wavelength LED used in each animal experiment (Figures 2D, 4A, 4D, and 4G). The minimization of Equation 4 allowed us to estimate the μ-opic sensitivity *s*_*μ*_(*λ*) shown in Figure 4K:

(Equation 5) $$s_{\mu }\left(\lambda \right)=\left\{ \begin{array}{c} \exp\left(-{\left(\frac{\lambda -429.583}{20.483}\right)}^{2}\right),\text{if}\;\lambda <429.583\;\text{nm}\\ \exp\left(-{\left(\frac{\lambda -429.583}{30.103}\right)}^{2}\right),\text{otherwise} \end{array}\right.$$

Figure S6 shows the photopic sensitivity *V*(*λ*) [-], the five action spectra *s*_*α*_(*λ*) [-] of the α-opic metric,^74^ and the here-obtained μ-opic sensitivity *s*_*μ*_(*λ*) [-]. The peak sensitivity the μ-opic occurs at a lower wavelength (430 nm) than all α-opic spectra including the S-cone-opic. While we assume a proportional relationship between efficacy and irradiance in Equations 3 and 4, a similar assumption can be made using the photon flux of each light source to derive the μ-opic sensitivity *s*_*p*,*μ*_(*λ*) [-] in the photon system:

(Equation 6) $$s_{p,\mu }\left(\lambda \right)=\left\{ \begin{array}{c} \exp\left(-{\left(\frac{\lambda -428.955}{20.413}\right)}^{2}\right),\text{if}\;\lambda <428.955\;\text{nm}\\ \exp\left(-{\left(\frac{\lambda -428.955}{29.746}\right)}^{2}\right),\text{otherwise} \end{array}\right.$$

With the μ-opic spectral sensitivity at hand, the tools developed for the α-opic metric can be employed to assess lighting conditions for myopia suppression by simply using the μ-opic sensitivity *s*_*μ*_(*λ*) instead of *s*_*α*_(*λ*) or *s*_*p*,*μ*_(*λ*) instead of *s*_*p*,*α*_(*λ*) [-] when working in the photon system. Table S1 illustrates μ-opic quantities that can be used for this purpose. We have employed the μ-opic equivalent luminance, μ-opic equivalent equi-energy illuminance, and μ-opic photon flux in Figures 5S–5Y to evaluate the myopia suppression characteristics of simulated lighting scenarios.

### Quantification and statistical analysis

Data were analyzed and presented using one of two distinct eye grouping structures: grouping structure A that encompassed various visual conditions, categorized into three subgroups: (i) normal visual experience, −10D lens, and no lens, (ii) different primary light sources (fluorescent and warm white), and (iii) supplemental narrowband LEDs with peak wavelengths of 366 nm, 395 nm, 400 nm, 410 nm, 421 nm, 432 nm, 441 nm, 454 nm, and 468 nm. Grouping structure B that was defined by combining the supplemental narrowband LED lights into broader spectral categories: UVA (366 nm, 395 nm), violet (400 nm, 410 nm), indigo (421 nm, 432 nm, 441 nm), and blue (454 nm, 468 nm). The left and right eyes of normal subjects showed no significant differences and were thus averaged. For longitudinal data analysis, grouping structure B was applied when the longitudinal trends observed in grouping structure A closely overlapped (UVA, indigo, blue), whereas grouping structure A was retained when the trends remained distinct (violet). Group differences in longitudinal data were assessed using a hierarchical model-building approach. Briefly, a linear mixed-effects model, incorporating linear and quadratic terms and time-group interaction effects, was fitted to the longitudinal data while accounting for correlated measurements. The necessity of quadratic terms was evaluated using a likelihood ratio test, which indicated a significant improvement in parameter estimates compared to a model with only linear terms. To assess the joint effect of the time-group interaction terms, a Wald chi-square test was conducted to simultaneously evaluate the significance of both the linear and quadratic coefficients on the model’s outcome. The treatment efficacy of the different supplemental short-wavelength lights was evaluated using refraction and axial length measurements obtained at the experimental endpoint (60 DVEs). Efficacy was calculated separately for refraction and axial length, and the two values were averaged for each eye. An efficacy of 0% and 100% corresponded to the average efficacy of eyes of the warm white light group with a −10 D lens and with no lens (control eye), respectively. Group differences in treatment efficacy were analyzed using a one-way analysis of variance (ANOVA) model. The fundamental assumptions of the ANOVA model, including normality and homoscedasticity, were verified using the Shapiro-Wilk test and Levene’s test before conducting the analysis. Data analysis and visualization were conducted using MATLAB R2021b, while statistical analyses were conducted in R Version 4.3.2, with a significance level of 0.05 applied to all tests.

## Supplemental information

Document S1. Figures S1–S8 and Table S1

Data S1. The file Grytz_et_al_2026_source_data.xlsx contains the source data used in this study Tabs within the file are as follows: (A) Light wavelength transmission data for the tree shrew lens. (B) Wavelength absorption data for tree shrew OPN5. (C) Irradiance data for LEDs used in this study. (D) Refraction and axial length data. (E) Efficacy data. (F) The μ-opic metric.

Document S2. Article plus supplemental information

## References

[1] Schaeffel F., Swiatczak B. (2024). Mechanisms of emmetropization and what might go wrong in myopia. Vision Res..

[2] Terakita A., Nagata T. (2014). Functional Properties of Opsins and their Contribution to Light-Sensing Physiology. Zool. Sci. (Tokyo).

[3] Park H.n., Jabbar S.B., Tan C.C., Sidhu C.S., Abey J., Aseem F., Schmid G., Iuvone P.M., Pardue M.T. (2014). Visually-driven ocular growth in mice requires functional rod photoreceptors. Investig. Ophthalmol. Vis. Sci..

[4] Chakraborty R., Yang V., Park H.N., Landis E.G., Dhakal S., Motz C.T., Bergen M.A., Iuvone P.M., Pardue M.T. (2019). Lack of cone mediated retinal function increases susceptibility to form-deprivation myopia in mice. Exp. Eye Res..

[5] Ji S., Mao X., Zhang Y., Ye L., Dai J. (2021). Contribution of M-opsin-based color vision to refractive development in mice. Exp. Eye Res..

[6] Linne C., Mon K.Y., D’Souza S., Jeong H., Jiang X., Brown D.M., Zhang K., Vemaraju S., Tsubota K., Kurihara T. (2023). Encephalopsin (OPN3) is required for normal refractive development and the GO/GROW response to induced myopia. Mol. Vis..

[7] Chakraborty R., Landis E.G., Mazade R., Yang V., Strickland R., Hattar S., Stone R.A., Iuvone P.M., Pardue M.T. (2022). Melanopsin modulates refractive development and myopia. Exp. Eye Res..

[8] Liu A.-L., Liu Y.-F., Wang G., Shao Y.-Q., Yu C.-X., Yang Z., Zhou Z.-R., Han X., Gong X., Qian K.-W. (2022). The role of ipRGCs in ocular growth and myopia development. Sci. Adv..

[9] Jiang X., Pardue M.T., Mori K., Ikeda S.I., Torii H., D’Souza S., Lang R.A., Kurihara T., Tsubota K. (2021). Violet light suppresses lens-induced myopia via neuropsin (OPN5) in mice. Proc. Natl. Acad. Sci. USA.

[10] Buhr E.D., Yue W.W.S., Ren X., Jiang Z., Liao H.-W.R., Mei X., Vemaraju S., Nguyen M.-T., Reed R.R., Lang R.A. (2015). Neuropsin (OPN5)-mediated photoentrainment of local circadian oscillators in mammalian retina and cornea. Proc. Natl. Acad. Sci..

[11] Nguyen M.-T.T., Vemaraju S., Nayak G., Odaka Y., Buhr E.D., Alonzo N., Tran U., Batie M., Upton B.A., Darvas M. (2019). An Opsin 5-dopamine pathway mediates light-dependent vascular development in the eye. Nat. Cell Biol..

[12] Yamashita T., Ono K., Ohuchi H., Yumoto A., Gotoh H., Tomonari S., Sakai K., Fujita H., Imamoto Y., Noji S. (2014). Evolution of mammalian Opn5 as a specialized UV-absorbing pigment by a single amino acid mutation. J. Biol. Chem..

[13] Kojima D., Mori S., Torii M., Wada A., Morishita R., Fukada Y. (2011). UV-sensitive photoreceptor protein OPN5 in humans and mice. PLoS One.

[14] A Second Revolution in Light and Health (2026) https://read.nxtbook.com/sage/lda/april_2026/a_second_of_revolution_in_light_and_health.html.

[15] Kondo S., Jiang X., Torii H., Mori K., Negishi K., Kurihara T., Tsubota K. (2025). Violet Light Is Abundant Outdoors but Deficient Indoors in Modern Lifestyle in Tokyo. Int. J. Environ. Res. Publ. Health.

[16] Jung B., Cheng Z., Brennan M., Inanici M., Pan Y., Yan D. (2023). Proceedings of Building Simulation 2023: 18th Conference of IBPSA.

[17] Holden B.A., Fricke T.R., Wilson D.A., Jong M., Naidoo K.S., Sankaridurg P., Wong T.Y., Naduvilath T.J., Resnikoff S. (2016). Global Prevalence of Myopia and High Myopia and Temporal Trends from 2000 through 2050. Ophthalmology.

[18] Houser K.W., Heschong L., Lang R.A. (2023). Buildings, lighting, and the myopia epidemic. LEUKOS - J. Illum. Eng. Soc. N. Am..

[19] Dolgin E. (2015). The myopia boom. Nature.

[20] Dolgin E. (2024). A myopia epidemic is sweeping the globe. Here’s how to stop it. Nature.

[21] Klepeis N.E., Nelson W.C., Ott W.R., Robinson J.P., Tsang A.M., Switzer P., Behar J.V., Hern S.C., Engelmann W.H. (2001). The National Human Activity Pattern Survey (NHAPS): a resource for assessing exposure to environmental pollutants. J. Expo. Anal. Environ. Epidemiol..

[22] KhalafAllah M.T., Fuchs P.A., Nugen F., El Hamdaoui M., Levy A., Redden D.T., Samuels B.C., Grytz R. (2023). Longitudinal Changes of Bruch’s Membrane Opening, Anterior Scleral Canal Opening, and Border Tissue in Experimental Juvenile High Myopia. Investig. Ophthalmol. Vis. Sci..

[23] Xiong L.-L., Sun Y.-F., Niu R.-Z., Xue L.-L., Chen L., Huangfu L.-R., Li J., Wang Y.-Y., Liu X., Wang W.-Y. (2024). Cellular Characterization and Interspecies Evolution of the Tree Shrew Retina across Postnatal Lifespan. Research.

[24] Hahn J., Monavarfeshani A., Qiao M., Kao A.H., Kölsch Y., Kumar A., Kunze V.P., Rasys A.M., Richardson R., Wekselblatt J.B. (2023). Evolution of neuronal cell classes and types in the vertebrate retina. Nature.

[25] Siegwart J.T., Norton T.T. (1998). The susceptible period for deprivation-induced myopia in tree shrew. Vision Res..

[26] Von Dollen P., Pimputkar S., Speck J.S. (2014). Let There Be Light-With Gallium Nitride: The 2014 Nobel Prize in Physics. Angew. Chem. Int. Ed. Engl..

[27] Chakraborty R., Ostrin L.A., Nickla D.L., Iuvone P.M., Pardue M.T., Stone R.A. (2018). Circadian rhythms, refractive development, and myopia. Ophthalmic Physiol. Opt..

[28] Henriksson J.T., Bergmanson J.P.G., Walsh J.E. (2010). Ultraviolet radiation transmittance of the mouse eye and its individual media components. Exp. Eye Res..

[29] Dillon J., Zheng L., Merriam J.C., Gaillard E.R. (1999). The optical properties of the anterior segment of the eye: implications for cortical cataract. Exp. Eye Res..

[30] Artigas J.M., Felipe A., Navea A., Fandiño A., Artigas C. (2012). Spectral transmission of the human crystalline lens in adult and elderly persons: color and total transmission of visible light. Investig. Ophthalmol. Vis. Sci..

[31] BOETTNER E.A., WOLTER J.R. (1962). Transmission of the Ocular Media. Investig. Ophthalmol. Vis. Sci..

[32] Brainard G.C., Beacham S., Sanford B.E., Hanifin J.P., Streletz L., Sliney D. (1999). Near ultraviolet radiation elicits visual evoked potentials in children. Clin. Neurophysiol..

[33] Kessel L., Lundeman J.H., Herbst K., Andersen T.V., Larsen M. (2010). Age-related changes in the transmission properties of the human lens and their relevance to circadian entrainment. J. Cataract Refract. Surg..

[34] Obana A., Gohto Y., Asaoka R. (2021). Macular pigment changes after cataract surgery with yellow-tinted intraocular lens implantation. PLoS One.

[35] McDowell R.J., Didikoglu A., Woelders T., Gatt M.J., Moffatt F., Notash S., Hut R.A., Brown T.M., Lucas R.J. (2024). Beyond Lux: methods for species and photoreceptor-specific quantification of ambient light for mammals. BMC Biol..

[36] Ho C.-L., Wu W.-F., Liou Y.M. (2019). Dose–Response Relationship of Outdoor Exposure and Myopia Indicators: A Systematic Review and Meta-Analysis of Various Research Methods. Int. J. Environ. Res. Publ. Health.

[37] Shah R.L., Huang Y., Guggenheim J.A., Williams C. (2017). Time Outdoors at Specific Ages During Early Childhood and the Risk of Incident Myopia. Investig. Ophthalmol. Vis. Sci..

[38] Ward G.J. (1994). In: Glassner AS, editor. Proceedings of the 21st annual conference on Computer graphics and interactive techniques SIGGRAPH ’94.

[39] Nazari M., Matusiak B., Stefani O. (2023). Utilising spectral lighting simulation technique for evaluating transmitted daylight through glazing: Exploring the non-visual effects and colour appearance. Heliyon.

[40] Pierson C., Aarts M.P.J., Andersen M. (2023). Validation of spectral simulation tools in the context of ipRGC-influenced light responses of building occupants. J. Build. Perform. Simul..

[41] Morgan I.G., French A.N., Ashby R.S., Guo X., Ding X., He M., Rose K.A. (2018). The epidemics of myopia: Aetiology and prevention. Prog. Retin. Eye Res..

[42] Morgan I., Rose K. (2005). How genetic is school myopia?. Prog. Retin. Eye Res..

[43] Tedja M.S., Haarman A.E.G., Meester-Smoor M.A., Kaprio J., Mackey D.A., Guggenheim J.A., Hammond C.J., Verhoeven V.J.M., Klaver C.C.W., CREAM Consortium (2019). IMI – Myopia Genetics Report. Investig. Ophthalmol. Vis. Sci..

[44] He X., Sankaridurg P., Wang J., Chen J., Naduvilath T., He M., Zhu Z., Li W., Morgan I.G., Xiong S. (2022). Time Outdoors in Reducing Myopia: A School-Based Cluster Randomized Trial with Objective Monitoring of Outdoor Time and Light Intensity. Ophthalmology.

[45] Xiong S., Sankaridurg P., Naduvilath T., Zang J., Zou H., Zhu J., Lv M., He X., Xu X. (2017). Time spent in outdoor activities in relation to myopia prevention and control: a meta-analysis and systematic review. Acta Ophthalmol..

[46] Pärssinen O., Lassila E., Kauppinen M. (2022). Associations of Children’s Close Reading Distance and Time Spent Indoors with Myopia, Based on Parental Questionnaire. Children.

[47] Torii H., Mori K., Okano T., Kondo S., Yang H.-Y., Yotsukura E., Hanyuda A., Ogawa M., Negishi K., Kurihara T., Tsubota K. (2022). Short-Term Exposure to Violet Light Emitted from Eyeglass Frames in Myopic Children: A Randomized Pilot Clinical Trial. J. Clin. Med..

[48] Torii H., Ohnuma K., Kurihara T., Tsubota K., Negishi K. (2017). Violet Light Transmission is Related to Myopia Progression in Adult High Myopia. Sci. Rep..

[49] Torii H., Kurihara T., Seko Y., Negishi K., Ohnuma K., Inaba T., Kawashima M., Jiang X., Kondo S., Miyauchi M. (2017). Violet Light Exposure Can Be a Preventive Strategy Against Myopia Progression. EBioMedicine.

[50] Mori K., Torii H., Hara Y., Hara M., Yotsukura E., Hanyuda A., Negishi K., Kurihara T., Tsubota K. (2021). Effect of Violet Light-Transmitting Eyeglasses on Axial Elongation in Myopic Children: A Randomized Controlled Trial. J. Clin. Med..

[51] Jeong H., Kurihara T., Jiang X., Kondo S., Ueno Y., Hayashi Y., Lee D., Ikeda S.-I., Mori K., Torii H. (2023). Suppressive effects of violet light transmission on myopia progression in a mouse model of lens-induced myopia. Exp. Eye Res..

[52] Wang Y., Ding H., Stell W.K., Liu L., Li S., Liu H., Zhong X. (2015). Exposure to sunlight reduces the risk of myopia in rhesus monkeys. PLoS One.

[53] Upton B.A., Díaz N.M., Gordon S.A., Van Gelder R.N., Buhr E.D., Lang R.A. (2021). Evolutionary Constraint on Visual and Nonvisual Mammalian Opsins. J. Biol. Rhythms.

[54] D’Souza S.P., Swygart D.I., Wienbar S.R., Upton B.A., Zhang K.X., Mackin R.D., Casasent A.K., Samuel M.A., Schwartz G.W., Lang R.A. (2021). Retinal patterns and the cellular repertoire of neuropsin (Opn5) retinal ganglion cells. J. Comp. Neurol..

[55] Sugihara T., Nagata T., Mason B., Koyanagi M., Terakita A. (2016). Absorption characteristics of vertebrate non-visual opsin, Opn3. PLoS One.

[56] Nayak G., Zhang K.X., Vemaraju S., Odaka Y., Buhr E.D., Holt-Jones A., Kernodle S., Smith A.N., Upton B.A., D’Souza S. (2020). Adaptive Thermogenesis in Mice Is Enhanced by Opsin 3-Dependent Adipocyte Light Sensing. Cell Rep..

[57] Sato M., Tsuji T., Yang K., Ren X., Dreyfuss J.M., Huang T.L., Wang C.-H., Shamsi F., Leiria L.O., Lynes M.D. (2020). Cell-autonomous light sensitivity via Opsin3 regulates fuel utilization in brown adipocytes. PLoS Biol..

[58] He L., Frost M.R., Siegwart J.T., Norton T.T. (2018). Altered gene expression in tree shrew retina and retinal pigment epithelium produced by short periods of minus-lens wear. Exp. Eye Res..

[59] Palczewski K. (2012). Chemistry and biology of vision. J. Biol. Chem..

[60] Neitz M., Wagner-Schuman M., Rowlan J.S., Kuchenbecker J.A., Neitz J. (2022). Insight from OPN1LW Gene Haplotypes into the Cause and Prevention of Myopia. Genes.

[61] Strickland R., Landis E.G., Pardue M.T. (2020). Short-Wavelength (Violet) Light Protects Mice From Myopia Through Cone Signaling. Investig. Ophthalmol. Vis. Sci..

[62] Eltanahy A.M., Aupetit A., Buhr E.D., Van Gelder R.N., Gonzales A.L. (2024). Light-sensitive Ca2+ signaling in the mammalian choroid. Proc. Natl. Acad. Sci. USA.

[63] Ostrin L.A., Harb E., Nickla D.L., Read S.A., Alonso-Caneiro D., Schroedl F., Kaser-Eichberger A., Zhou X., Wildsoet C.F. (2023). IMI—The Dynamic Choroid: New Insights, Challenges, and Potential Significance for Human Myopia. Investig. Ophthalmol. Vis. Sci..

[64] Khanal S., Norton T.T., Gawne T.J. (2023). Limited bandwidth short-wavelength light produces slowly-developing myopia in tree shrews similar to human juvenile-onset myopia. Vision Res..

[65] Niwa H., Yamamura K., Miyazaki J. (1991). Efficient selection for high-expression transfectants with a novel eukaryotic vector. Gene.

[66] Siegwart J.T., Norton T.T. (1994). Goggles for controlling the visual environment of small animals. Lab. Anim. Sci..

[67] Norton T.T., Wu W.W., Siegwart J.T. (2003). Refractive state of tree shrew eyes measured with cortical visual evoked potentials. Optom. Vis. Sci..

[68] El Hamdaoui M., Gann D.W., Norton T.T., Grytz R. (2019). Matching the LenStar optical biometer to A-Scan ultrasonography for use in small animal eyes with application to tree shrews. Exp. Eye Res..

[69] Webber J.B.W. (2012). A bi-symmetric log transformation for wide-range data. Meas. Sci. Technol..

[70] Govardovskii V.I., Fyhrquist N., Reuter T., Kuzmin D.G., Donner K. (2000). In search of the visual pigment template. Vis. Neurosci..

[71] Lamb T.D. (1995). Photoreceptor spectral sensitivities: common shape in the long-wavelength region. Vision Res..

[72] Yamashita T., Ohuchi H., Tomonari S., Ikeda K., Sakai K., Shichida Y. (2010). Opn5 is a UV-sensitive bistable pigment that couples with Gi subtype of G protein. Proc. Natl. Acad. Sci. USA.

[73] Abboushi B., Safranek S., Davis R., Kim J.K., Krames M.R., Strassburg M. (2021). Light-Emitting Devices, Materials, and Applications XXV Proceedings of SPIE.

[74] Lucas R.J., Peirson S.N., Berson D.M., Brown T.M., Cooper H.M., Czeisler C.A., Figueiro M.G., Gamlin P.D., Lockley S.W., O’Hagan J.B. (2014). Measuring and using light in the melanopsin age. Trends Neurosci..

