# Supplementary material for: Prevention of myopia in a near-primate by supplemental indigo light suggests a hypothesis for the myopia boom
Source: Cell Rep Med. 2026 Aug 18;7(9):102999. doi: 10.1016/j.xcrm.2026.102999 (PMC13589487; doi:10.1016/j.xcrm.2026.102999)
Supplement: Supplementary file 1 [file mmc1.pdf]

Cell Reports Medicine, Volume *7*

Supplemental information

Prevention of myopia in a near-primate by supplemental indigo light suggests a hypothesis for the myopia boom

Rafael Grytz, Mustapha El Hamdaoui, Takahiro Yamashita, Shane P. D'Souza, Mahmoud Tawfik KhalafAllah, Mehlika Inanici, and Richard A. Lang

Supplemental Information

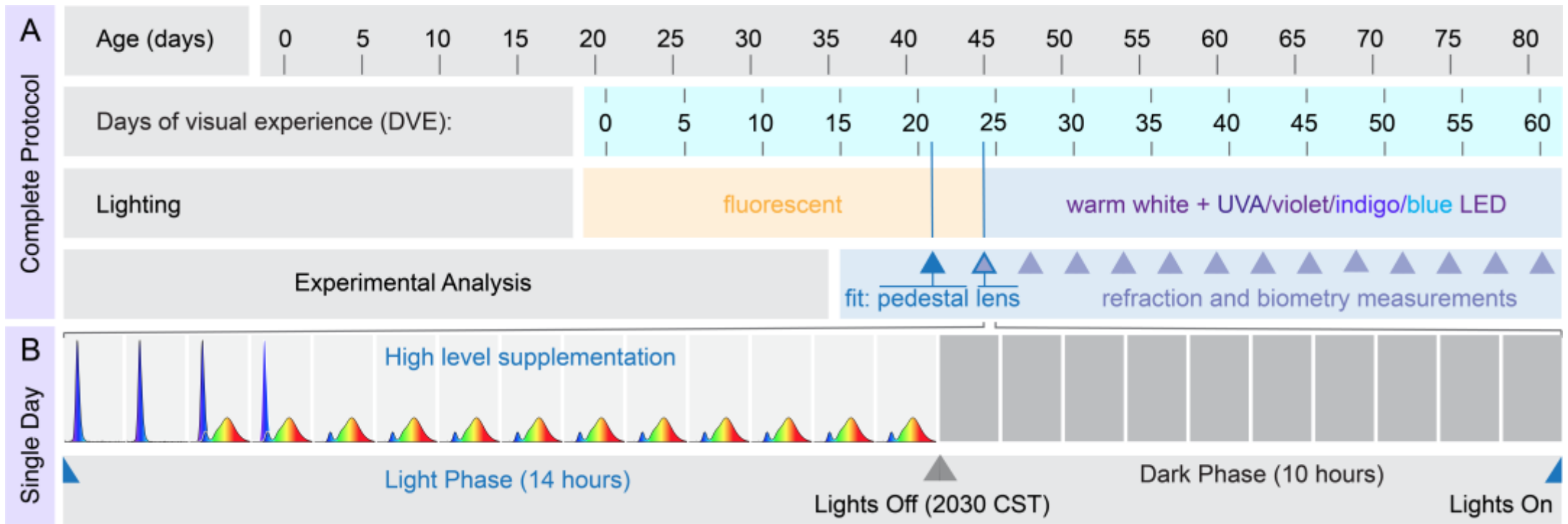

Supplemental Figure 1. The protocol for induced myopia in the tree shrew

Related to Figs. 2, 4 and STAR Methods. (A) Schematic aligning tree shrew age (top row), with the days of visual experience (DVE) after the animals open their eyes at approximately day 21 (second row), with the lighting (third row). This includes standard colony fluorescent lighting or experimental lighting, to which the animals were exposed. Shown in the fourth row is the experimental analysis including fitting of the head pedestal and lens, with refraction and biometry measurements taken from DVE24 to DVE60.

(B) Schematic showing the experimental lighting protocol for a single day. Short wavelength UVA, violet, indigo or blue wavelengths were used to supplement the light produced by a warm white LED. Supplemental wavelengths were turned on for four hours at the beginning of the 14-hour light phase to mimic the spectral distribution of a natural dawn on a summer day. LED: Light emitting diode. CST: Central Standard Time.

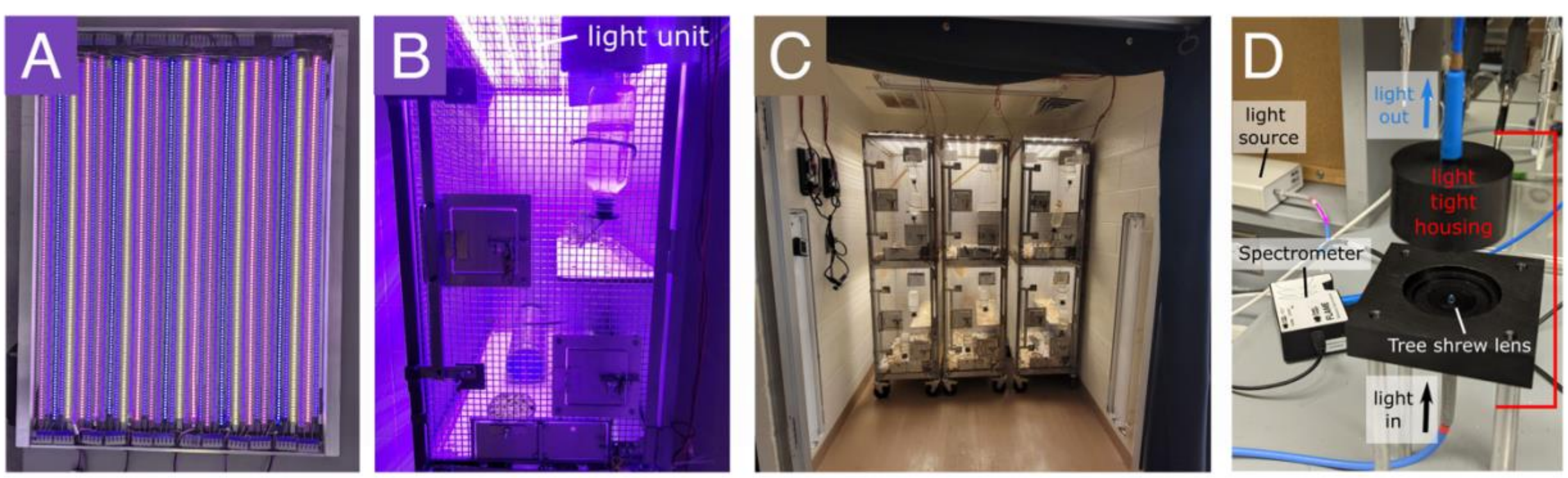

Supplemental Figure 2. Equipment for controlled lighting and lens transmission quantification

Related to Figs. 2-4 and STAR Methods. (A) Image showing one light unit for controlled lighting experiments. Each unit incorporates warm white (3000k) and narrowband short wavelength LEDs, independently controlled using pulse wave modulation. (B) Image showing one tree shrew cage with the light unit on top.

(C) Image showing one bay with six cages with individual light units. Only animals with the same lighting conditions were housed in the same bay. Each bay was isolated from other light sources using blackout curtains.

(D) Image showing the optic fiber-coupled measurement device used for assessing the light transmission characteristics of the tree shrew lens. The device consists of a full spectrum light source coupled to a light-tight housing that positions the ocular lens within the light path, and a downstream spectrometer for measurements.

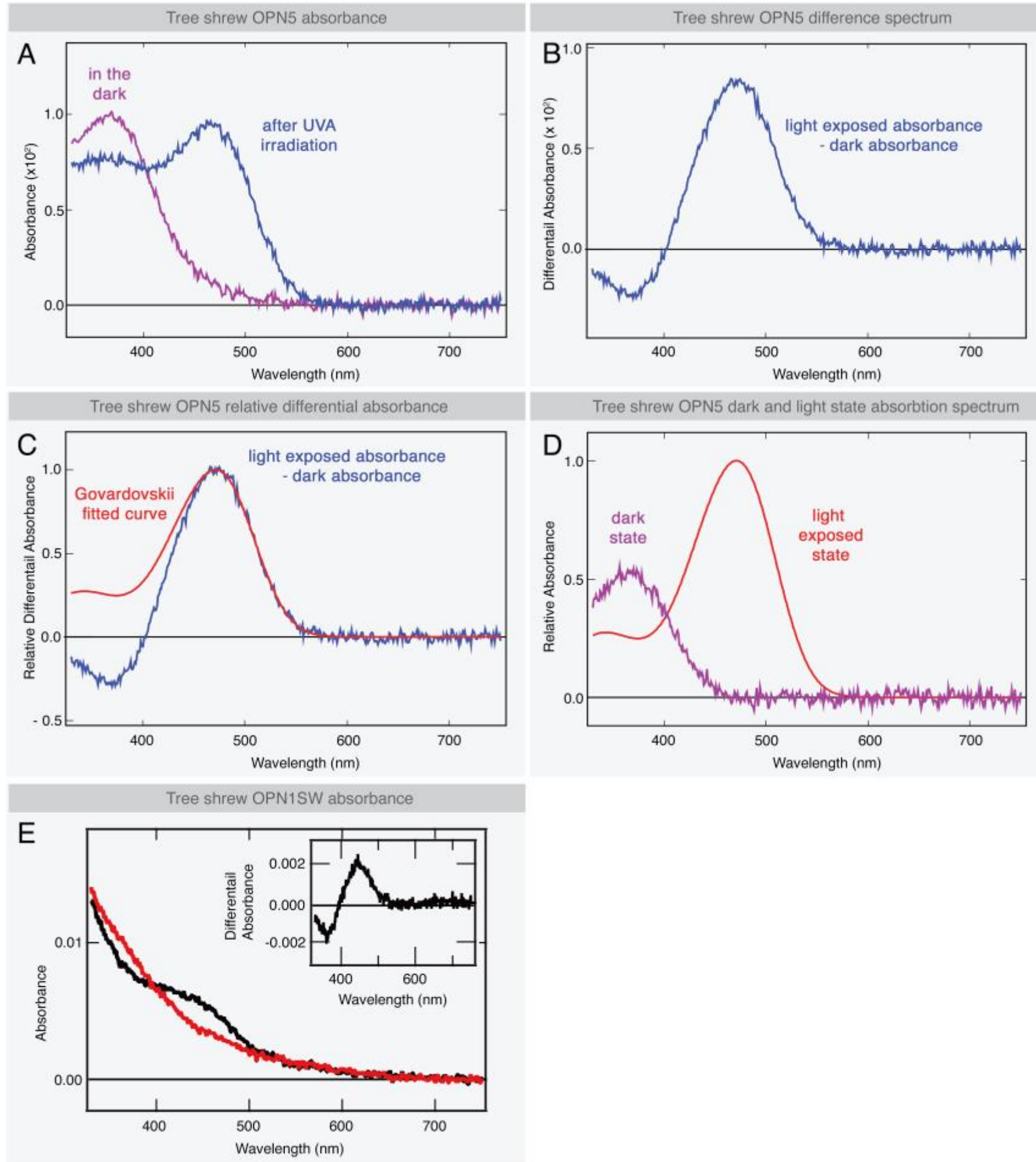

Supplemental Figure 3: Tree shrew OPN5 and OPN1SW absorption spectra

Related to Fig. 3. (A) Absorption spectra of *Tupaia belangeri chinensis* OPN5 in the dark (purple curve) and after UVA irradiation (blue curve).

(B) Difference spectrum of OPN5 obtained by subtracting the spectrum before irradiation (purple curve in a) from that measured after UV light irradiation (blue curve in A).

(C) Absorption spectrum (blue curve) of all-*trans* retinal-bound state of OPN5. The red curve is the fitted spectrum according to Govardovskii.

(D) Absorption spectrum (purple curve) of 11-*cis* retinal-bound state of OPN5. Red curve as in (C). The purple curve is obtained by subtracting blue curve in c from red curve in c.

(E) The absorption spectrum for *Tupaia belangeri chinensis* OPN1SW. Recombinant protein of OPN1SW was purified after the addition of 11-*cis* retinal and their absorption spectra were measured in the dark (black curve) and after yellow light (>460 nm) irradiation (red curve) in the presence of 5mM NH_2OH. (Inset) Spectral change caused by yellow light irradiation. The positive peak at around 435 nm corresponds to the absorption peak of OPN1SW.

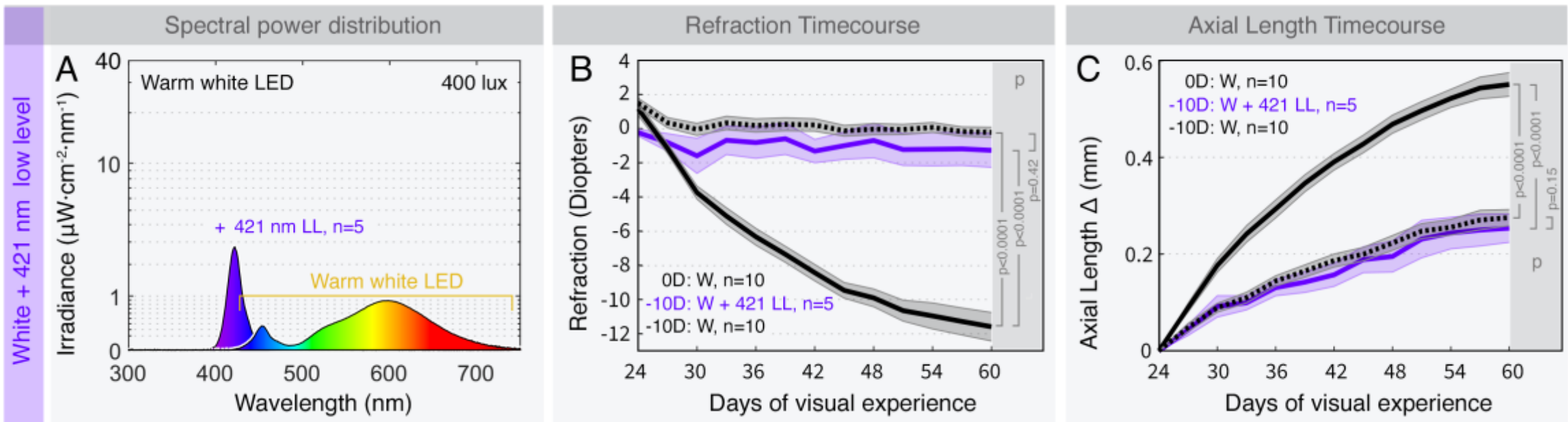

Supplemental Figure 4: Low level indigo supplementation effectively suppresses induced myopia

Related to Figs. 4 and 5. (A) Chart showing spectral power output from warm white LED (orange bracket) and the supplemental low-level (LL) indigo light with a peak at 421 nm.
(B, C) Charts showing refraction (B) and axial length (C) for eyes in warm white LED lighting with no lens (0D: W, n=10, dashed black traces) or with a -10 Diopter lens (-10D: W, n=10, solid black traces), or with warm white LED and 421 nm supplementation and a -10D lens (-10D: W + 421, n=5, purple traces).

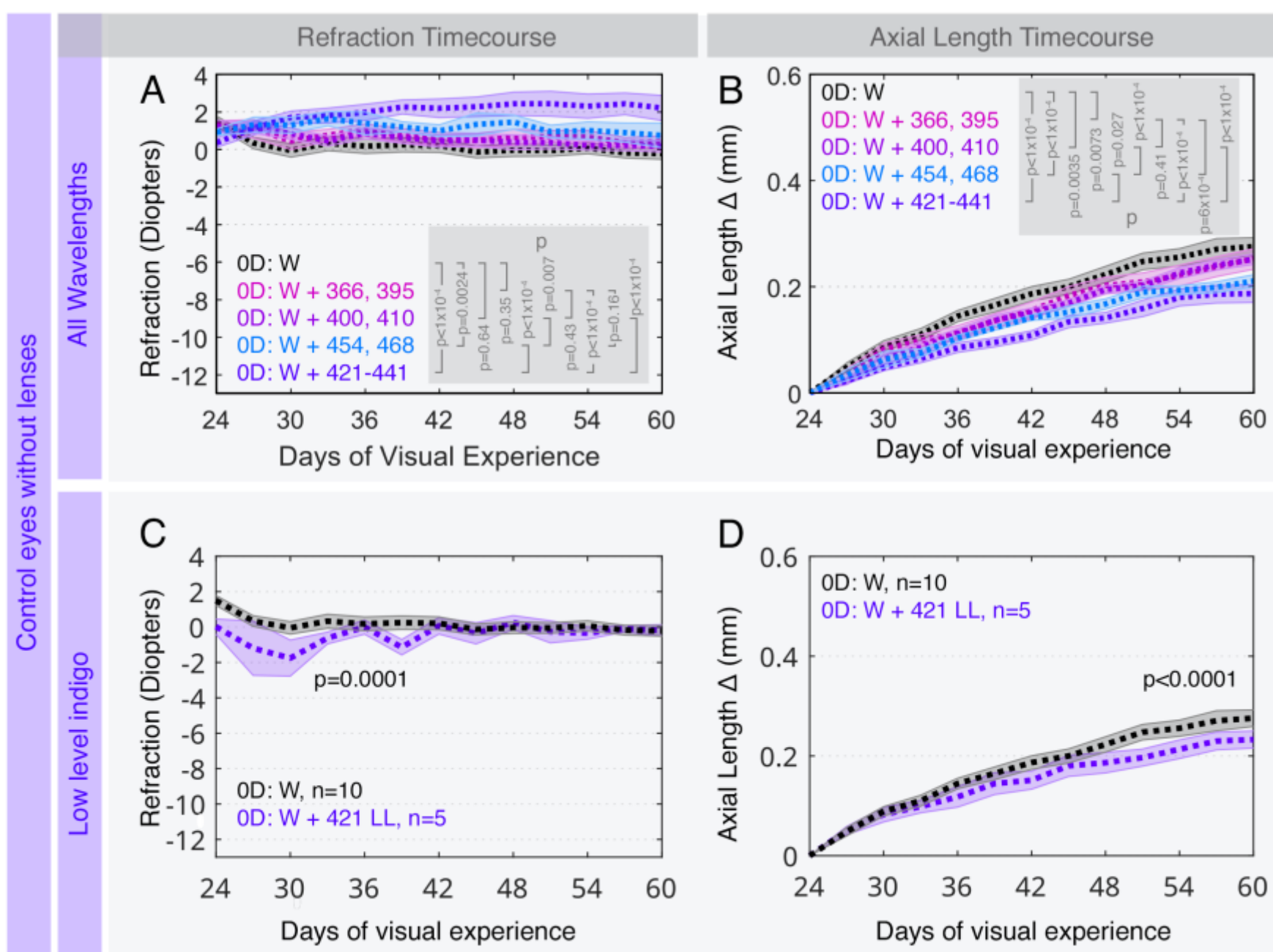

Supplemental Figure 5: Refraction and axial length in control eyes with no lens

Related to Figs. 2 and 4. (A, B) Refraction (A) and axial length (B) for all control eyes with no lens exposed to warm white LED light without short wavelength supplementation (0D; W) or with supplementation (0D:W+366,395; 0D:W+400, 410; 0D:W+454,468; 0D:W+421-441) according to the spectra defined in (Fig. 4 A, D and G). p-values comparing the different control eye cohorts are shown on the grey shaded region within the charts. (C, D) Refraction (C) and axial length (D) for control eyes with no lens exposed to warm white LED light without short wavelength supplementation (0D; W, n=10) or with low level indigo supplementation (0D:W+421 LL) according to the spectra defined in Supplemental Fig. 4A. p-values are shown

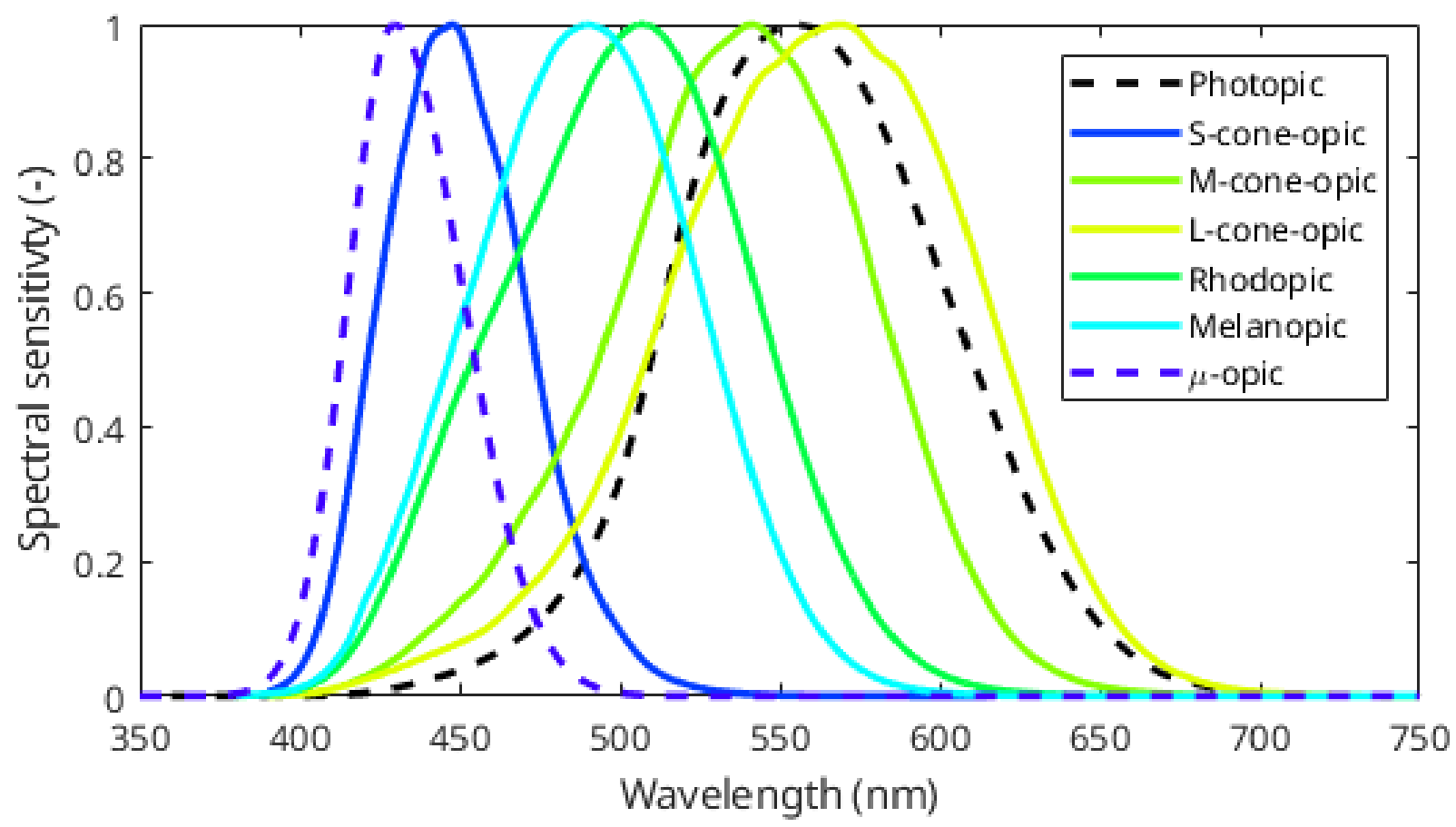

Supplemental Figure 6.
Related to Fig. 5. The µ-opic spectral sensitivity $s_\mu(\lambda)$, photopic sensitivity V(λ), and the five action spectra of the α-opic metrics $s_\alpha(\lambda)$ representing the spectral sensitivity of S-cone, M-cone, L-cone, Rhodopsin and Melanopsin, all normalized to have a maximum value of 1.

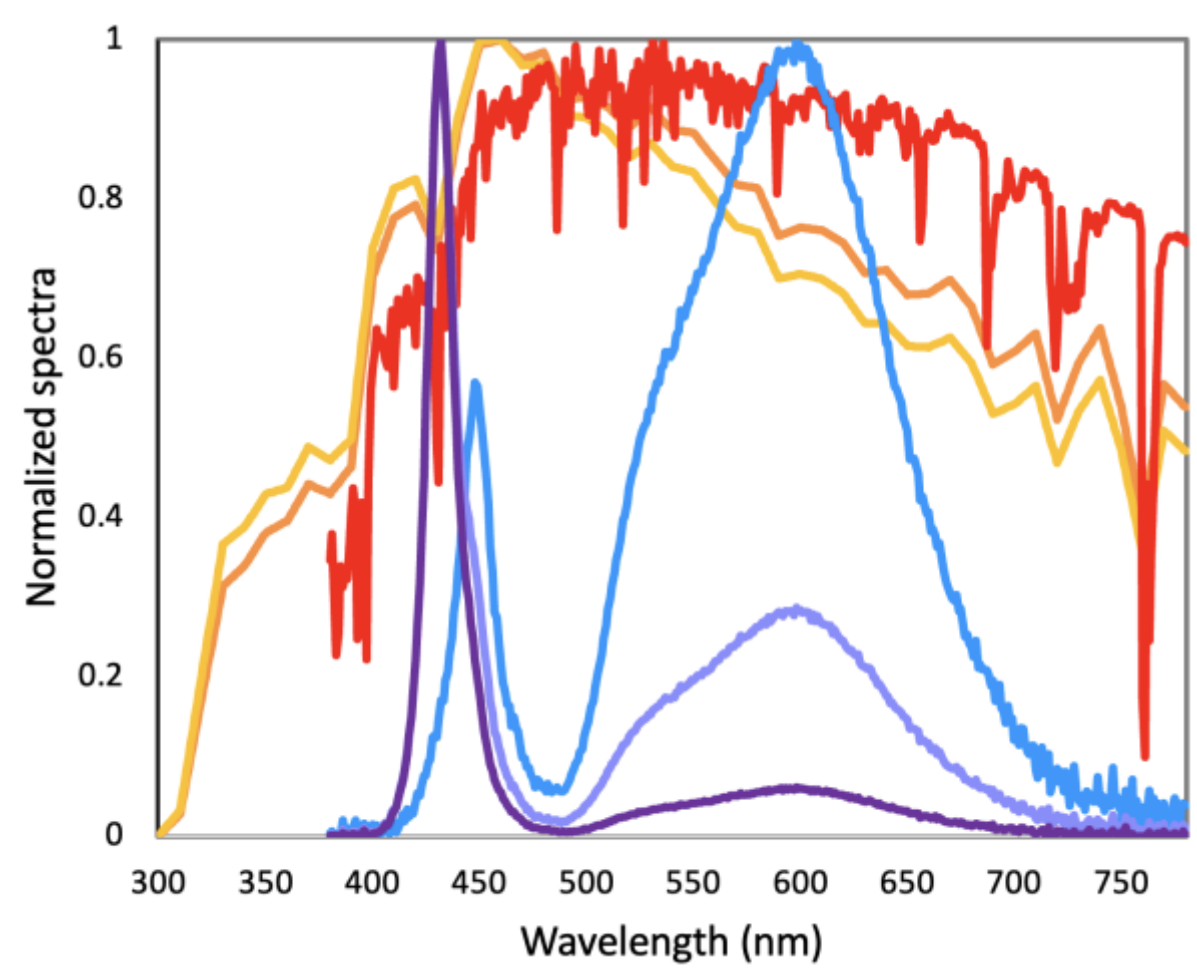

Supplemental Figure 7.
Related to Fig. 5. Each trace represents illumination source spectra normalized to a maximum value of 1 for the conditions described. Red trace: Direct irradiance (sun) with the spectral properties as specified in ASTM G173. Orange trace: 6500K correlated color temperature simulated for June 21st at noon with a direct normal irradiance (DNI) of 328 W/m^2 and diffuse horizontal irradiance (DHI of 296 W/m^2. Yellow trace: 7000K correlated color temperature overcast sky conditions simulated for December 28 at noon, with DNI of 0 (no direct sunlight) and DHI of 29 W/m^2. Blue trace: Normalized spectral output of the warm white light emitting diode lighting condition (WW LED). Indigo trace: Normalized spectral output of the high-level indigo supplemented warm white LED lighting condition (WW LED + 421-441). Violet trace: Normalized spectral output of the low-level indigo supplemented warm white LED lighting condition (WW LED + 421 LL).

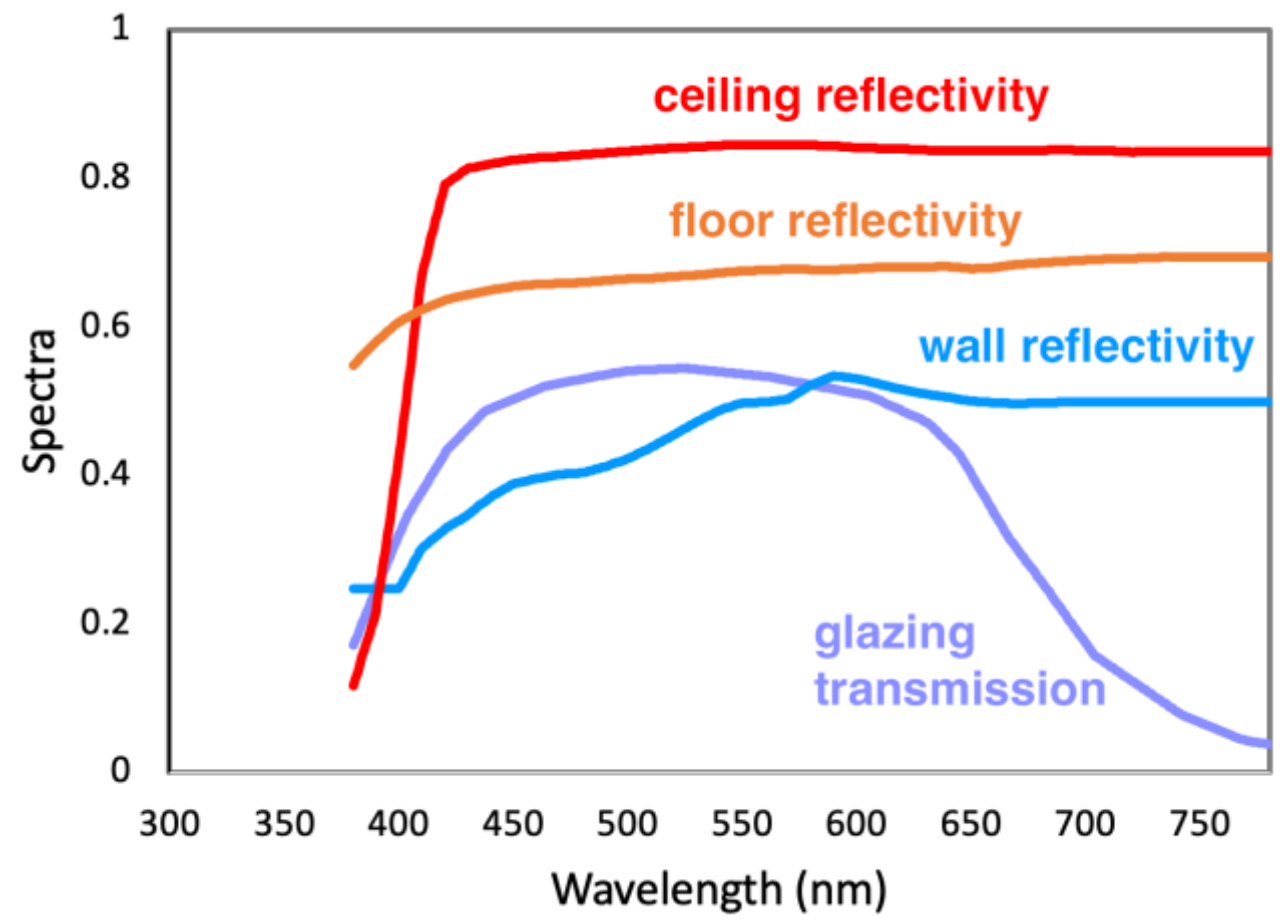

Supplemental Figure 8.
Related to Fig. 5. The spectral properties of major surfaces used in the simulated classroom. Red trace: Reflectivity of the ceiling. Orange trace: Reflectivity of the floor. Indigo trace: Transmission of the glazing. Blue trace: Reflectivity of the wall.

Quantity	Formula	Meaning	Unit
**µ-opic irradiance**	$E_\mu = \int E_{e,\lambda}(\lambda) \cdot s_\mu(\lambda)\, d\lambda$	effective photobiological irradiance to suppress myopia	W·m^{-2}
**µ-opic efficacy of luminous radiation**	$K_{\mu,v} = \frac{\int \phi_{e,\lambda}(\lambda) \cdot s_\mu(\lambda)\, d\lambda}{K_m \int \phi_{e,\lambda}(\lambda) \cdot V(\lambda)\, d\lambda}$	quotient of µ-opic radiant flux and luminous flux	W·lm^{-1}
**µ-opic equivalent daylight (D65) illuminance (µ-EDI)**	$E_{v,\mu}^{\mathrm{D65}} = \frac{E_\mu}{K_{\mu,v}^{\mathrm{D65}}}$ $K_{\mu,v}^{\mathrm{D65}} = 0.6207$ mW·lm^{-1}	Illuminance level of standard daylight (D65) that provides an equal µ-opic irradiance as the test source	lx
**µ-opic equivalent equi-energy illuminance (Eq. µ-opic lux)**	$E_{v,\mu}^{\mathrm{E}} = K_{N,\mu}\, E_\mu$	Illuminance of equi-energy (E) radiation required to provide equal µ-opic irradiance as the test source	lx
**µ-opic equivalent illuminance constant**	$K_{N,\mu} = K_m \frac{\int E_{e,\lambda}^{\mathrm{E}}(\lambda)\, V(\lambda)\, d\lambda}{\int E_{e,\lambda}^{\mathrm{E}}(\lambda) s_\mu(\lambda)\, d\lambda}$	For $E_{e,\lambda}^{\mathrm{E}}(\lambda) = 1$ this constant is $K_{N,\mu} =$ 1627.991 lm·W^{-1}	lm·W^{-1}
**µ-opic equivalent illuminance conversion**	$E_{v,\mu}^{\mathrm{E}} = 1.0105 \cdot E_{v,\mu}^{\mathrm{D65}}$	Conversion between µ-opic equivalent daylight (D65) and equivalent equi-energy (E) illuminances	lx
**µ-opic equivalent luminance**	$L_{v,\mu}^{\mathrm{E}} = \frac{L_\alpha}{K_{\mu,v}^{E}}$	Luminance of equi-energy (E) radiation required to provide equal µ-opic irradiance as the test source	candela·m^{-2}
**µ-opic photon flux**	$\phi_{p,\mu} = \int \phi_{p,\lambda}(\lambda) \cdot s_{p,\mu}(\lambda)\, d\lambda$	effective photobiological photon flux to suppress myopia	photons cm^{-2}·sec^{-1}

Supplemental Table 1.

Related to Fig. 4. Example of µ-opic quantities to characterize short wavelength light for its ability to suppress myopia. λ – wavelength [nm], $E_{e,\lambda}(\lambda)$ – spectral irradiance [W·m^{-2}·nm^{-1}], $s_\mu(\lambda)$ – µ-opic spectral sensitivity [-], $\phi_{e,\lambda}(\lambda)$ – spectral radiant flux [W·nm^{-1}], $K_m = 683.002$ lm·W^{-1} – maximum spectral luminous efficacy, $V(\lambda)$ – photopic sensitivity [-], $K_{\mu,v}^{\mathrm{D65}}$ – µ-opic efficacy of luminous radiation of daylight (D65), $\phi_{p,\lambda}(\lambda)$ – spectral photon flux [photons cm^{-2}·sec^{-1}·nm^{-1}], $s_{p,\mu}(\lambda)$ – µ-opic spectral sensitivity in the photon system [-].
